# Modeling 1-Cyano-4-Dimethylaminopyridine Tetrafluoroborate (CDAP) Chemistry to Design Glycoconjugate Vaccines with Desired Structural and Immunological Characteristics

**DOI:** 10.3390/vaccines12070707

**Published:** 2024-06-24

**Authors:** Rebecca Nappini, Renzo Alfini, Salvatore Durante, Laura Salvini, Maria Michelina Raso, Elena Palmieri, Roberta Di Benedetto, Martina Carducci, Omar Rossi, Paola Cescutti, Francesca Micoli, Carlo Giannelli

**Affiliations:** 1 Dipartimento di Scienze della Vita, Università Degli Studi di Trieste, Via L Giorgieri 1, Ed. C11, 34127 Trieste, Italy; rebecca.x.nappini@gsk.com (R.N.); pcescutti@units.it (P.C.); 2 GSK Vaccines Institute for Global Health (GVGH), 53100 Siena, Italy; renzo.x.alfini@gsk.com (R.A.); salvatore.x.durante@gsk.com (S.D.); maria-michelina.m.raso@gsk.com (M.M.R.); elena.x.palmieri@gsk.com (E.P.); roberta.x.di-benedetto@gsk.com (R.D.B.); martina.x.carducci@gsk.com (M.C.); omar.x.rossi@gsk.com (O.R.); francesca.x.micoli@gsk.com (F.M.); 3 Fondazione Toscana Life Sciences (TLS), 53100 Siena, Italy; l.salvini@toscanalifesciences.org

**Keywords:** 1-cyano-4-dimethylaminopyridine tetrafluoroborate (CDAP), conjugation, design of experiment, *S.* Paratyphi A, 1,4-diazabicyclo[2.2.2]octane, DABCO, lutidine

## Abstract

Glycoconjugation is a well-established technology for vaccine development: linkage of the polysaccharide (PS) antigen to an appropriate carrier protein overcomes the limitations of PS T-independent antigens, making them effective in infants and providing immunological memory. Glycoconjugate vaccines have been successful in reducing the burden of different diseases globally. However, many pathogens still require a vaccine, and many of them display a variety of glycans on their surface that have been proposed as key antigens for the development of high-valency glycoconjugate vaccines. CDAP chemistry represents a generic conjugation strategy that is easily applied to PS with different structures. This chemistry utilizes common groups to a large range of PS and proteins, e.g., hydroxyl groups on the PS and amino groups on the protein. Here, new fast analytical tools to study CDAP reaction have been developed, and reaction conditions for PS activation and conjugation have been extensively investigated. Mathematical models have been built to identify reaction conditions to generate conjugates with wanted characteristics and successfully applied to a large number of bacterial PSs from different pathogens, e.g., *Klebsiella pneumoniae*, *Salmonella* Paratyphi A, *Salmonella* Enteritidis, *Salmonella* Typhimurium, *Shighella sonnei* and *Shigella flexneri*. Furthermore, using *Salmonella* Paratyphi A O-antigen and CRM_197_ as models, a design of experiment approach has been used to study the impact of conjugation conditions and conjugate features on immunogenicity in rabbits. The approach used can be rapidly extended to other PSs and accelerate the development of high-valency glycoconjugate vaccines.

## 1. Introduction

Bacterial polysaccharides (PSs) have proven to be optimal targets for vaccine development. They are made by repeating units constituted of monosaccharides linked together by glycosidic linkages and surround the surfaces of many bacteria, helping them to survive in unpredictable and often hostile environments [1]. Chemical conjugation of PS antigens to appropriate carrier proteins is an established procedure for improving their immunogenicity, thus overcoming limitations of T-independent PS, e.g., lack of memory and no efficacy in infants [2]. Glycoconjugate vaccines have been licensed against *N. meningitidis*, *H. influenzae*, *St. pneumoniae* and *S.* Typhi and have proven to be safe and effective [3]. Nevertheless, additional glycoconjugates are needed to extend the coverage of certain vaccines, as in the case of *St. pneumoniae* [4], or to address other emerging serotypes (i.e., *N. meningitidis* serogroup X or *H. influenzae* type a). Furthermore, alarming concern is emerging regarding antimicrobial resistant (AMR) bacteria, which are directly responsible for 1.27 million deaths per year and mainly affect low- and middle-income countries [5]. Vaccines represent a major tool to fight AMR; they work prophylactically, reducing the number of infectious disease cases, antibiotic use and spread of AMR [6]. Among the top 10 causes of global deaths attributable to and associated with bacterial AMR, vaccines are available only against *S. pneumoniae* and *Mycobacterium tuberculosis*. Due to the variety of glycan antigens displayed on the surfaces of pathogens for which vaccines are still not available (e.g., *E. coli*, *S. aureus*, *K. pneumoniae*, *Shigella*, *S.* Paratyphi A), potent high-valency glycoconjugate vaccines are a desirable target. Affordability and rapid manufacturing remain one of the limitations of current glycoconjugates.

Traditionally, conjugate vaccines are prepared by PS extraction from bacterial fermentation, subsequent purification and chemical binding to the carrier protein [7]. Since very often native PSs have a rather large molecular weight (MW), they are subjected to fragmentation and sizing to obtain shorter saccharides of defined MW range, whose use improves process robustness and simplifies manufacturing and analytical characterization [7,8]. The carbohydrate moiety is chemically linked to the carrier protein, and two main approaches can be used: one based on PS coupling to the protein through activation of the terminal group of the sugar chain and the other based on chemical activation of multiple groups along the PS chain followed by conjugation. Appropriate linkers are often used to increase conjugation efficiency by reducing steric hindrance between the two conjugate components, PS and the carrier protein. The conjugation chemistry, the carrier protein used, the saccharide loading and the saccharide chain length are all parameters that can affect the immunogenicity of glycoconjugate vaccines and need to be optimized for the design of an effective glycoconjugate [3]. Different chemistries have been proposed to prepare conjugate vaccines, selected on the basis of the specific structure of the PS used [2,9].

**Figure 1:**
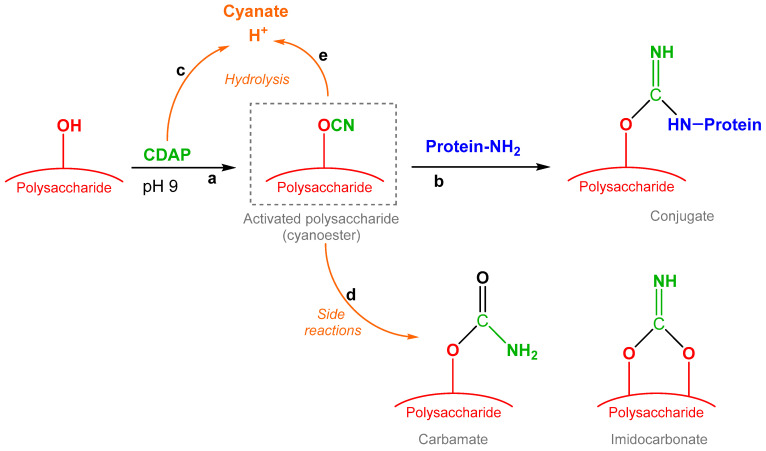
Activation and conjugation processes of PS via CDAP chemistry. Activation and conjugation are indicated by paths **a** and **b**, respectively, while **c**, **d** and **e** show side reactions in which both PS, CDAP and activated PS can be involved.

Lees identified 1-cyano-4-dimethylaminopyridinium tetrafluoroborate (CDAP) chemistry as a fast, simple, one-step conjugation strategy. After PS activation with CDAP, which is able to randomly activate the hydroxyl groups along the PS chain through a cyanylation reaction, the carrier protein is directly added to the mixture without isolation of the activated PS as an intermediate [10,11]. No use of linkers is required, thus avoiding additional derivatization steps for the carrier protein or the PS. The activated PS reacts with amino groups commonly present in proteins (Figure 1).

Activation of the PS and conjugation efficiently occur at pH 9; the PS activation step is fast (<3 min) and requires a real-time pH adjustment as CDAP hydrolysis lowers the pH. Typically, more than 90% of CDAP is hydrolyzed during the reaction, generating an equal molar amount of H^+^; for example 1 mg/mL CDAP corresponds to ~4 mM, while at pH 9, OH^−^ is 0.01 mM. This makes the process difficult to control and challenging to scale up. Triethylamine is the base generally employed for the adjustment of pH.

The control of reaction conditions is important to limit the hydrolysis of the CDAP reactant and of the activated PS as well as the possible side reactions resulting in byproducts such as carbamate and imidocarbonate (IMC) (Figure 1).

To improve the manufacturability and the reproducibility of the reaction, two main improvements were proposed in 2020:

1. The use of 4-Dimethylaminopyridine HCl (DMAP) as buffer during PS activation: DMAP is compatible with CDAP but considering its pKa (9.7) [12], it has a limited buffering capacity at the target pH, so there is still the need for real-time pH adjustment (performed with sodium hydroxide). However, DMAP’s presence limits the risk of pH overshooting during adjustments. Several other buffers have been tried, but all of them resulted in lower activation efficiency [13], probably due to direct reaction with CDAP.
2. Performing the reaction at 0 °C in an ice bath instead of 25 °C: as the CDAP reaction kinetic is too fast and influenced by temperature, this method results in better temperature control and easier pH adjustment, as the reaction lasts 15 min instead of 3 min at this lower temperature.

In this work, we have further developed the protocol of CDAP activation/conjugation chemistry to make it more robust and consistent, and we identified which reaction conditions are critical parameters and how they impact the quality attributes of the final conjugates. Furthermore, statistical tools were used to support the rapid design of conjugates with different structural features and understand optimal glycoconjugate design for an increased immune response. The chemistry used and further developed here can be rapidly extended to PSs having different structures, supporting the development of high-valency glycoconjugate vaccines, which are of great interest for global health.

## 2. Materials and Methods

### 2.1. Reagents

The following chemicals were used: KDO, CDAP, DABCO, DMAP, Lutidine, Dextran 25 kDa and 150 kDa, Adipic acid dihydrazide (ADH), 2-(N-morpholino)ethanesulfonic acid (MES), N-etil-N′-(3-dimetilaminopropyl)carbodiimide (EDAC), cysteine and sodium pyruvate from Sigma-Aldrich (St. Louis, MO, USA). *Salmonella*, *Shigella* and *Klebsiella* OAg were purified as previously described [14,15]. *Klebsiella* strains for CPS isolation were cultured on a carbohydrate-rich Worfel–Ferguson agar medium and CPS purified as previously described [16]. The Vi polysaccharide was purified as previously described [17]. CRM_197_ (58.4 kDa) was obtained from GSK R&D (Siena, Italy).

CDAP solutions were prepared at 100 mg/mL in acetonitrile (ACN). The DABCO solution was prepared at a 0.5 M concentration in water, adding 1 M HCl to reach pH 9.2 (this resulted in pH 9.0 after 10-fold dilution of the solution). The 0.5 M DMAP solution was prepared in water, adding 1 M HCl to reach pH 9.2 (this resulted in pH 9.0 after 10-fold dilution of the solution). The Lutidine solution was prepared at 0.5 M concentration in water, adding 1 M HCl to reach pH 7.2 (this resulted in pH 7.0 after 10-fold dilution of the solution).

CRM_197_ was prepared in saline with a 5 mM DABCO buffer at about 20 mg/mL concentration by buffer exchange using Amicon 10K (Merck, Darmstadt, Germany) centrifugation devices (2 × 10 min at 4000 rcf).

### 2.2. Software

Design Expert 13 (StatEase, Inc., Minneapolis, MN, USA) was used for DoE planning and elaboration; Minitab 18.1 (Minitab, LLC, State College, PA, USA) was used for all other statistical analyses.

### 2.3. PS Activation

The PS was solubilized in water (with the eventual addition of NaCl) at the specified concentration. Under magnetic stirring (in an ice bath at 0 °C, 5 °C or 25 °C), DABCO 0.5 M was added to reach a 50 mM concentration at pH 9 (alternative buffers/pHs were used in some experiments: 2,6-Lutidine/1-methylimidazole, N-methylmorpholine, DMAP). The CDAP 100 mg/mL solution in ACN was then added to have the desired reactant quantity. After the desired activation time (typically 15 min), an aliquot of the solution was analyzed for calculating the PS activation degree, and the rest was used for conjugation.

For kinetic studies, at different time points starting from the CDAP addition, an aliquot of the reaction mixture was analyzed as described in Section 2.4.

### 2.4. PS Activation Degree by HPLC-SEC Analysis

An aliquot of the activated PS solution was quenched by the addition of an equal volume of 0.5 M ADH in water and maintained for 2 h at 25 °C. Excess of ADH present in the sample was reduced with a PD10 (Cytiva, Chicago, IL, USA) disposable desalting column. The column was pre-equilibrated with 2 M NaCl. The sample was diluted with 4 M NaCl to 1.5 mL total volume and loaded on the column with stack procedure as reported in the PD10 product manual, adding immediately after, 1 mL of 4 M NaCl to reach a total volume of 2.5 mL of loaded solution on the column. The sample was finally eluted with 3.5 mL of water. After removal of the majority of ADH excess, the PS-ADH sample (100 μL), with a concentration in terms of hydrazide groups between 15.7 nmol/mL and 156.7 nmol/mL, was added to 100 μL of pyruvate solution (13.7 mg sodium pyruvate dissolved in 5 mM sodium acetate, pH 3.8). Underivatized sample was also prepared and run as a blank: 100 μL of the PS-ADH sample at the same dilution used for the analysis was added to 100 μL of water.

The calibration curve was built with KDO standards (100 μL) in the range of 15.7 nmol/mL–156.7 nmol/mL, and 100 μL of a semicarbazide solution (100 mg semicarbazide hydrochloride + 90.5 mg of sodium acetate in 10 mL of water) was added. All samples and standards were heated at 50 °C for 50 min and then analyzed by HPLC-SEC (80 μL injection) on a TSKgel 3000 PW-XL column (Tosoh Bioscience GmbH, Griesheim, Germany) using 0.1 M NaCl, 0.1 M sodium phosphate at pH 7.2 containing 5% ACN as eluent at a flow rate of 0.5 mL/min.

For quantification of the activated groups, 252 nm UV ABS is used for detection. The area of the peak corresponding to the pyruvate derivatized PS-ADH was corrected by subtracting the area of the corresponding blank (if not negligible) and used for the quantification with respect to the calibration curve obtained with semicarbazide-KDO standard peak areas.

For PS quantification, a refractive index detector was used. The area of the PS-ADH sample blank was measured for the quantification with respect to a standard PS curve typically in the range of 31.25–500 μg/mL.

The activation degree was then calculated from the nmol/mL of hydrazide in PS-ADH and saccharide amount, considering the MW of the repeating unit of the PS, and expressed as the molar ratio between the ADH and saccharide rings or between the ADH and PS repeating unit.

### 2.5. Conjugation

After PS activation time, cooled CRM_197_ at about 20 mg/mL in a solution of 5 mM DABCO, 9 g/L NaCl at pH 9 was added to the reaction at 0 °C in order to achieve the desired PS/CRM_197_ ratio. The reaction mixture was kept under mixing at the indicated temperature (0°, 5° or 25 °C) for the time needed (typically 25 °C for 2 h). After conjugation time, 1 M glycine in 50 mM sodium phosphate at pH 7 was added in equal volume with respect to the reaction mixture to quench eventual residual cyanoester groups, and the reaction mixture was kept at 2–8 °C overnight.

For kinetic studies, at different time points starting from the CRM_197_ addition, an aliquot of the reaction mixture was quenched with an equal volume of the glycine solution, kept at 2–8 °C overnight and analyzed.

### 2.6. CRM_197_-ADH Synthesis

CRM_197_ was derivatized with ADH as previously described [18]. Briefly, CRM_197_ was prepared at 10 mg/mL in an 80 mM MES buffer at pH 6 using an Amicon 10K (Merck) centrifugation device (2× 10 min at 4000 rcf). ADH (ADH/protein = 3.5 *w*/*w*) was added to CRM_197_ and, after its dissolution, EDAC was added (EDAC/protein = 0.15 *w*/*w*). The reaction was carried out for 1 h at RT; then the reaction mixture was exchanged with 5 mM DABCO, 1 M NaCl at pH 9 (9 × 10 min at 4000 rcf) followed by 5 mM DABCO 150 mM NaCl at pH 9 (9 × 10 min at 4000 rcf) using an Amicon 10K (Merck) centrifugation device.

### 2.7. Conjugates MW Determination

Conjugates were analyzed by HPLC-SEC (80 μL injection) on a TSKgel 5000 PW column (Tosoh Bioscience GmbH) using 0.1 M NaCl 0.1 M sodium phosphate at pH 7.2 containing 5% ACN as eluent at a flow rate of 0.5 mL/min. For MW determination, a refractive index detector with the dextran MW standard (Sigma-Aldrich) calibration curve (50, 80, 150, 270, 410 and 670 KDa) was used performing a linear regression between log(MW) and retention time. Data are reported as Mp (the mode of the molecular weight distribution).

### 2.8. OAg MW Determination

OAg was analyzed by HPLC-SEC (80 μL injection) on a TSKgel 3000 PW-XL column (Tosoh Bioscience GmbH) using 0.1 M NaCl 0.1 M sodium phosphate at pH 7.2 containing 5% ACN as eluent at a flow rate of 0.5 mL/min. For MW determination, a refractive index detector with the dextran MW standard (Sigma-Aldrich) calibration curve (5, 12, 25, 50, 80 and 150 KDa) was used performing a linear regression between log(MW) and retention time. Data are reported as Mp (the mode of the molecular weight distribution).

### 2.9. S. Paratyphi A OAg Quantification

OAg quantification was performed by measuring the rhamnose quantity with the Dische assay [19] considering the polysaccharide repeating unit MW. Briefly, 1050 μL of ice-cooled sulfuric acid was added to 500 μL of each standard or sample. The tubes were heated at 100 °C for 5 min and then cooled in ice for 10 min. One milliliter of each treated standard or sample was transferred into a disposable cuvette, and the ABS 427 nm and 396 nm were read. Thirty-two microliters of a solution of cysteine (Cys) 1 M was added to each cuvette, and the cuvettes were capped and mixed by vortexing. The cuvettes were then left for 10 min at RT for color development, and the ABS 427 nm and 396 nm were read. For each standard and sample, ΔABS was calculated using the following formula:

$$ABS={(ABS}_{396}^{Post\;Cys}-{ABS}_{396}^{Pre\;Cys})-{(ABS}_{427}^{Post\;Cys}-{ABS}_{427}^{Pre\;Cys})$$

The sample concentration was determined with respect to the calibration curve obtained by using a linear regression between ΔABS of the standards and their concentrations (in the range of 2–32 μg/mL of Rhamnose).

### 2.10. Protein Quantification

Protein quantification was performed with a micro-BCA assay (Pierce) according to the manufacturer’s instructions.

### 2.11. Free CRM_197_ Determination in Conjugates

Conjugates were analyzed by HPLC-SEC (10 μL injection) on a TSKgel 2000 SW-XL column (Tosoh Bioscience GmbH) using 0.1 M Na_2_SO_4_ 0.1 M sodium phosphate at pH 6.6 as eluent at a flow rate of 0.3 mL/min. Unconjugated CRM_197_ was quantified using a fluorimeter detector (280 nm excitation/336 nm emission) with a CRM_197_ standard calibration curve in the range of 10–100 μg/mL.

### 2.12. Free PS Quantification in Conjugates

First, 1 mL of the conjugate diluted to 100 μg/mL (in terms of protein) in 5 mM sodium phosphate at pH 7.2 with 150 mM NaCl and 0.005% Tween 20 was loaded on a C4 Solid Phase extraction disposable column (Vydac Bioselect C4, 100 mg/3 mL, Grace, Columbia, MD, USA). Solution was eluted by applying a positive air pressure over the liquid phase, and the flow through was collected. Then, 1 mL of 20% ACN in water with 0.05% TFA was loaded into the cartridge to maximize free PS recovery, and the eluate was collected together with the initial flow through. The entire 2 mL volume collected was dried with a centrifugal evaporator and re-suspended in a suitable volume of water to quantify the free saccharide by the Dische colorimetric assay [19].

For the DoE runs, the PS to protein ratio in the conjugates was calculated as the difference between the total saccharide and the residual free saccharide determined in the reaction mixture. In all tests, free CRM_197_ was not detected.

### 2.13. MS Analysis

First, 2.5 mg of the cyclodextrin sample (see Section 3.2) was added to sodium chloride to obtain a 4 M final concentration, and then the sample was desalted using PD MidiTrap G-10 (Cytiva) following the manufacturer’s protocol. Two microliters of the sample was mixed with 2 μL of a 5 mg/mL solution of 2,5-DHB in ACN/water (50/50, *v*/*v*). The resulting mixture was spotted on the MALDI target and left to dry in the air. The mass spectra were acquired over the mass range *m*/*z* of 1000–3000 Da using UltrafleXtreme (Bruker Daltonics, GmbH, Bremen, Germany) in positive reflectron mode. A peptide calibration mixture (Bruker Daltonics, GmBH) was used for calibration.

CRM_197_ samples (see Section 3.5) of about 5 μM concentration were analyzed on an Orbitrap Q-Exactive HF-X mass spectrometer (Thermofisher, Waltham, MA, USA) by direct flow injection at a flow rate of 8 µL/min with a syringe pump. The spectra were acquired in ESI-positive HMR mode using an extended mass range between *m*/*z* 1000 and 6000 at a resolution of 15 K at *m*/*z* 200. The spray voltage was set to 2.7 kV, capillary temperature to 320 °C and sheath gas (nitrogen) to 10 a.u.

### 2.14. DoE

The experiments were performed over 4 different days (blocks), each block containing a half fraction of the design and three center point replicates for a total of 28 runs, 7 per day (Figure S14 and Table S2 report a detailed list of the experiments performed). In order to ensure the stabilization of the pH in all the ranges of CDAP concentrations tested, a DABCO buffer at pH 9 was used at a final concentration of 100 mM. Based on the preliminary experiments, activation time/temperature and protein conjugation time/temperature were fixed respectively at 15′ at 0 °C and 2 h at 25 °C.

All the responses were measured for each run following the same randomization order used in the DoE plan (and same blocks, when needed).

To elaborate the data, for each response, a linear model with two-factor interaction terms was chosen, and the data were not transformed before analysis. Non-significant terms (*p*-value > 0.05) were removed from the model using a backward elimination process.

Prior to DoE execution, 28 Eppendorf tubes (2 mL) were filled using the same starting *S.* Paratyphi A OAg solution, each with the PS quantity needed for the respective DoE run, and dried overnight in a centrifugal evaporator (Table S2).

The following solutions were prepared and maintained at the indicated temperature: CDAP 100 mg/mL in acetonitrile (indicated in the experimental procedure below as *CDAP100*, −20 °C); 100 mM DABCO with 500 mM NaCl at pH 9.2 (*DABCO100*, 2–8 °C); 1 M glycine with 50 mM sodium phosphate at pH 7.2 (*glycine1M*, 2–8 °C); 500 mM ADH in water (*ADH500*, 2–8 °C); 154 mM NaCl with 5 mM DABCO at pH 9 (*DABCO5*, 2–8 °C); 20 mg/mL CRM_197_ in 154 mM NaCl with 5 mM DABCO at pH 9 (*CRM20*, 2–8 °C).

#### Experimental Procedure

Each day, the 7 tubes containing the dried PS for the respective runs were added with 1 mL of the *DABCO100* to redissolve the PS and kept under stirring in an ice bath at 0 °C. The vial containing the *CDAP100* was equilibrated at 0 °C, and the needed amount of CDAP solution was added to each tube (Table S2). The solution was maintained under stirring for 15 min.

Next, 200 μL of each reaction mixture was added to a tube containing 200 μL of *ADH500* in an ice bath and then maintained under stirring at 25 °C for 2 h. This mixture was then used to calculate the activation % of the PS following the analytical procedure reported above.

To the remaining 800 μL of the activated PS, the required amounts of the following pre-cooled solutions were immediately added in sequence: *DABCO5* and *CRM20* (as per details in Table S2). The reaction mixture was then maintained under stirring for 2 h at 25 °C and finally quenched by transferring each mixture in 5 mL tubes containing 1.4 mL of the *glycine1M* solution and maintained overnight under stirring at 2–8 °C.

Finally, each reaction mixture was desalted using a PD10 (Cytiva) disposable column exchanging with a sodium phosphate buffer 20 mM at pH 6.5. The conjugates were then subjected to quantification of total PS and total protein, conjugate MW, free CRM_197_ and free PS determination.

### 2.15. Immunogenicity Study in Rabbits

All animal sera used in this study were derived from rabbit immunization experiments performed at the Charles River Laboratories (France). Animal studies were reviewed by the local ethical committee (Project code: APAFIS #39244-2022110910396896 v3; Date of approval: 19 April 2023) and carried out in compliance with animal welfare standards according to European Directive 63/2010 and to local legislation.

In detail, eight female New Zealand White rabbits per group were injected intramuscularly (IM) two times with 500 μL/dose of 5 μg of O:2 at day 0 and 28 in the presence of Alhydrogel (0.375 mg/dose Al^3+^).

In all animal studies, bleeds were collected at day 28 (post I), and the final bleed was at day 42 (post II). Collected blood was left at room temperature for decantation for 30 min for serum separation. Afterwards, samples were centrifuged at 2800 rpm for 15 min at +5 ± 3 °C.

Anti-O:2 IgG response by ELISA and Serum Bactericidal Activity (SBA) were performed according to the procedures already published [20].

### 2.16. Formulation Preparation

Formulations were prepared by adding excipients and antigen in the following order: water, alhydrogel, a 100 mM phosphate buffer at pH 6.5, a 90 g/L saline solution and a O:2-CRM_197_ conjugate.

Formulations with O:2-CRM_197_ conjugates had an O:2 concentration of 10 μg/mL in the presence of Alhydrogel (0.75 mg/mL final Al^3+^ concentration), 20 mM of a phosphate buffer at pH 6.5 and 9 g/L NaCl. In this condition, O:2-CRM_197_ conjugates were adsorbed on Alhydrogel with a total unabsorbed protein content of 5–20% (quantified by micro-BCA).

### 2.17. Data Analysis

Multiple regression analyses (ANOVA, Design Expert software-version: 13.0.11.0) were performed on the log-transformed EU/mL (day 28 or day 42) and log-transformed SBA IC50 (day 42) in order to find the surface to correlate the log-transformed immunological data (ELISA or SBA) with DoE factors or conjugate characteristics. The interpolation was performed considering a surface with the first order terms and all the interactions between them (2FI model). Subsequently, an iterative backward elimination (with a threshold of *p* < 0.1) of non-significant terms was applied.

## 3. Results

### 3.1. A New HPLC-SEC Analysis for Determining PS Cyanylation Degree

As reported in the literature [10,13], cyanoesters introduced along the PS chains through CDAP activation can be quantified after quenching with adipic acid dihydrazide (ADH). Reaction of activated cyanylated hydroxyl groups with hydrazides is a fast, quantitative method and essentially independent from the pH [10]. After removal of the excess unreacted ADH through extensive dialysis, hydrazide groups introduced on the PS chain are quantified by TNBS (2,4,6-Trinitrobenzenesulfonic acid) colorimetric assay. The quantification of PS after dialysis is performed by colorimetric phenol sulfuric acid assay, and the level of activation is calculated in terms of hydrazide groups/saccharide ratio [11].

**Figure 2:**
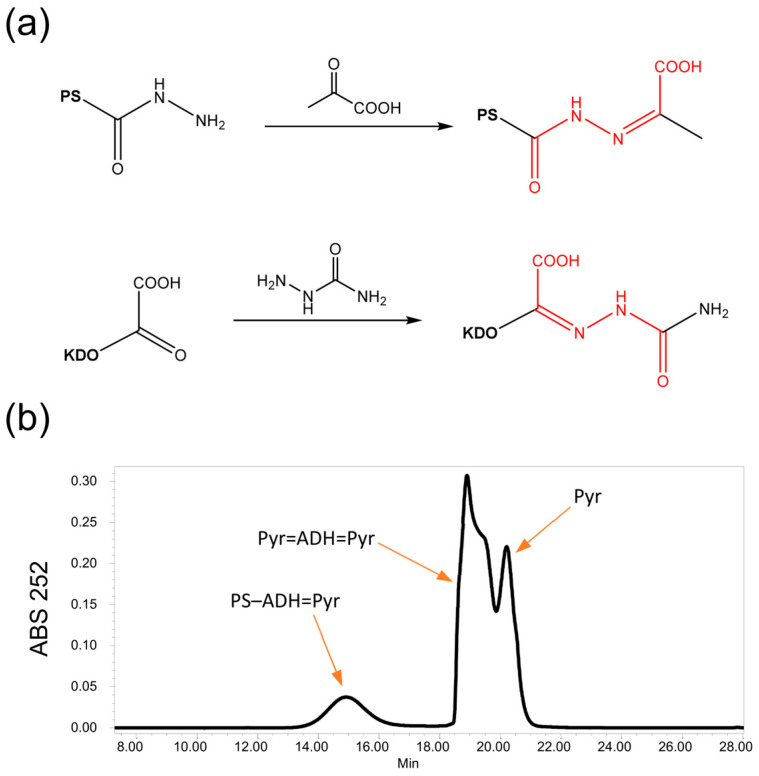
(**a**) Reaction scheme of PS-ADH with pyruvate and of KDO with semicarbazide resulting in the formation of the same chromophore (highlighted in red); (**b**) example of HPLC-SEC chromatographic profile of dextran-ADH derivatized with pyruvate. In the chromatogram PS-carrying ADH groups derivatized with pyruvate (PS–ADH=Pyr), residual free ADH present in the desalted sample derivatized with pyruvate (Pyr=ADH=Pyr) and excess of pyruvate reactant (Pyr) used for sample derivatization are evidenced.

We set up a new, faster analytical method for determining the degree of PS cyanylation by HPLC-SEC. Hydrazide groups introduced on the PS chain are derivatized with pyruvate (Pyr), and the resulting chromophore (α-ketohydrazone) is detected by UV absorption at the diagnostic wavelength of 252 nm (Figure 2). The quantification of introduced ADH is made by comparison of the activated PS peak area with a standard calibration curve obtained by reaction of standard 3-Deoxy-D-manno-oct-2-ulosonic acid (KDO) with semicarbazide resulting in a species with the same chromophore (α-ketohydrazone) (Figure 2) [19,21]. Standard ADH derivatized with pyruvate was not suitable to be used for the calibration curve as it produces a broad peak that partially overlaps with pyruvate’s residual peak (free pyruvate, due its high concentration in the reaction, also is visible at 252 nm ABS). On the other hand, KDO=semicarbazide has the same chromophore of ADH=Pyr and elutes in a clean zone of the chromatogram. Moreover, it has been verified that it has a comparable molar absorption with respect to the hydrazide-pyruvate moiety.

Clear advantages of this methodology with respect to the original one are (1) tagged PS is detected by faster analysis, avoiding extensive dialysis (reduction of free ADH by desalting with a disposable column is enough to perform the analysis); (2) only a small amount of the activated PS is needed for the test; (3) ADH introduced on the PS chain and PS are quantified at the same time by Abs at 252 nm (Pyr=ADH chromophore quantification) and by the refractive index (dRI) (PS quantification); and (4) possible changes in size of the PS (e.g., crosslinking, depolymerization) are monitored and can be quantified with an appropriate Gel Permeation Chromatography (GPC) standard calibration curve.

PS activation is calculated as nmol/mL ADH on nmol/mL of PS sugar rings or repeating units (RUs); the latter is useful to understand the extent of impact of the activation on PS epitopes.

### 3.2. Identification of a Novel Buffer System for Activation of PS with CDAP

We have introduced some modifications to the original protocol expanding the idea of the use of a buffer able to maintain a stable pH at 9 during the reaction without affecting the efficiency of the PS activation.

The use of DMAP (with a pKa of 9.7) still requires a pH adjustment to maintain the reaction at the target pH of 9 by promptly adding aliquots of 0.1 M NaOH after CDAP addition [13,22]. Almost all buffers having a pKa close to 9 contain a nucleophilic group like a primary amine that can react directly with the activated PS or hydroxyl groups that can be activated by CDAP. We introduced 1,4-diazabicyclo[2.2.2]octane HCl (DABCO) as an alternative buffer for the PS activation with CDAP. DABCO is a bicyclic tertiary amine that is widely used as a catalyst and reagent in polymerization and organic synthesis. DABCO, like DMAP, has no OH or primary/secondary amino groups that can react with CDAP or activated PS, but it has a higher buffer capacity at the target pH of 9, having a pKa of 8.7 [23]. In this way, we have verified that the pH is stable and its adjustment can be avoided during the reaction. DABCO is also a greener chemistry alternative product [24] with respect to DMAP, which is a highly toxic substance.

For starters, CDAP activation of a 25 kDa dextran as a model PS was performed in the presence of DABCO as the buffer compared to DMAP [13], keeping all other conditions identical. The activation degree calculated by HPLC-SEC (6.1% for DABCO and 6.8% for DMAP) showed no statistical difference (*n* = 3) between the two protocols for dextran activation (Figure S1). The DABCO protocol was also tested with 150 kDa dextran in the same conditions, resulting in a similar activation degree of 7.1%.

For some PSs, pH 9 could result in unwanted structural modification (e.g., depolymerization or decrease in O-acetylation). For this reason, we also identified an appropriate buffer system that can work at pH 7.

Different organic bases that seemed suitable to avoid self-reaction with CDAP or activated PS were evaluated: 2,6-lutidine (pKa 6.72 [25]), N-methylmorpholine (pKa 7.38 [26]) and 1-methylimidazole (pKa 7 [27]).

With an initial screening using *S.* Paratyphi A O-antigen (O:2) (4.4 mg/mL O:2, CDAP/OAg 0.5 *w*/*w*, pH 7, 4 h, 0 °C), the activation percentage using N-methylmorpholine (1.1%) was lower than by using 1-methylimidazole (3.9%) or 2,6-lutidine (4.2%). 2,6-lutidine was selected because of its lower toxicity with respect to 1-methylimidazole.

We also investigated the formation of eventual side products during the activation of PS with CDAP in a DABCO buffer [13,28] (Figure 1). γ-cyclodextrin (CD) was used as a model at this scope, being a monodisperse cyclic oligosaccharide allowing for easier mass spectroscopy analysis with respect to polydisperse PS samples. Activations were performed with different CDAP to PS ratios at pH 7 or 9, and the activated PSs were quenched with ADH. MS spectra clearly showed the sodium adduct peaks of unreacted cyclodextrin at *m*/*z* 1319.4 and dextrin linked with one or two ADH molecules at *m*/*z* 1518.5 and *m*/*z* 1717.5, respectively. Only imidocarbonate species at *m*/*z* 1362.4, 1543.5 and 1742.5, having none, one or two ADH introduced, respectively, were revealed as side products (Figure S2). With respect to what has been reported in the literature, no other undesired products were generated with CDAP chemistry in DABCO or a lutidine buffer.

The peak intensity ratios, proportional to quantity ratios, between the imidocarbonate side product and the corresponding ADH derivatized cyclodextrin were similar in all conditions tested (Table S1). Moreover, these ratios increased with the activation degree, which is represented (being proportional to) by the ratios between peak intensities of ADH-derivatized products and the unreacted cyclodextrin in Table S1.

### 3.3. Parameters Affecting PS Activation with CDAP

Some preliminary tests were performed using dextran 25 kDa and O:2 *S.* Paratyphi A O-antigen as models to identify the critical parameters affecting the activation degree; reaction time, temperature, pH, salt concentration, PS and CDAP concentrations were investigated.

**Figure 3:**
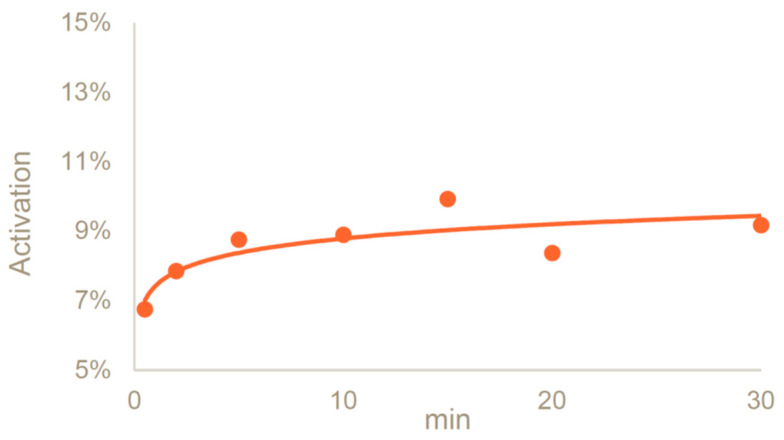
Activation kinetic of dextran 4.4 mg/mL with CDAP/PS 0.5 *w*/*w* in a DABCO buffer at pH 9 at 0 °C.

In the presence of DABCO, we evaluated the activation of dextran 25 kDa at different timepoints, performing the reaction at 0 °C up to 30 min. An activation degree close to 9% was reached after 5 min of reaction, and the resulting activated dextran was stable for at least 30 min (9.2%) (Figure 3).

The conditions identified (CDAP/PS ratio of 0.5 *w*/*w* in the presence of DABCO as a buffer for an activation time of 15 min at 0 °C) were applied to *S.* Paratyphi A OAg by working with three different starting OAg concentrations of 4.4, 2.2 and 1.1 mg/mL. While activation resulted in 7.5% per ring at both 4.4 mg/mL and 2.2 mg/mL PS concentrations, at 1.1 mg/mL, the activation decreased to 3.8%.

The impact of CDAP/PS ratio was also investigated by working with dextran and *S.* Paratyphi A OAg (Table 1). For both PSs, the activation degree increased by working with higher CDAP/PS ratios.

**Figure 4:**
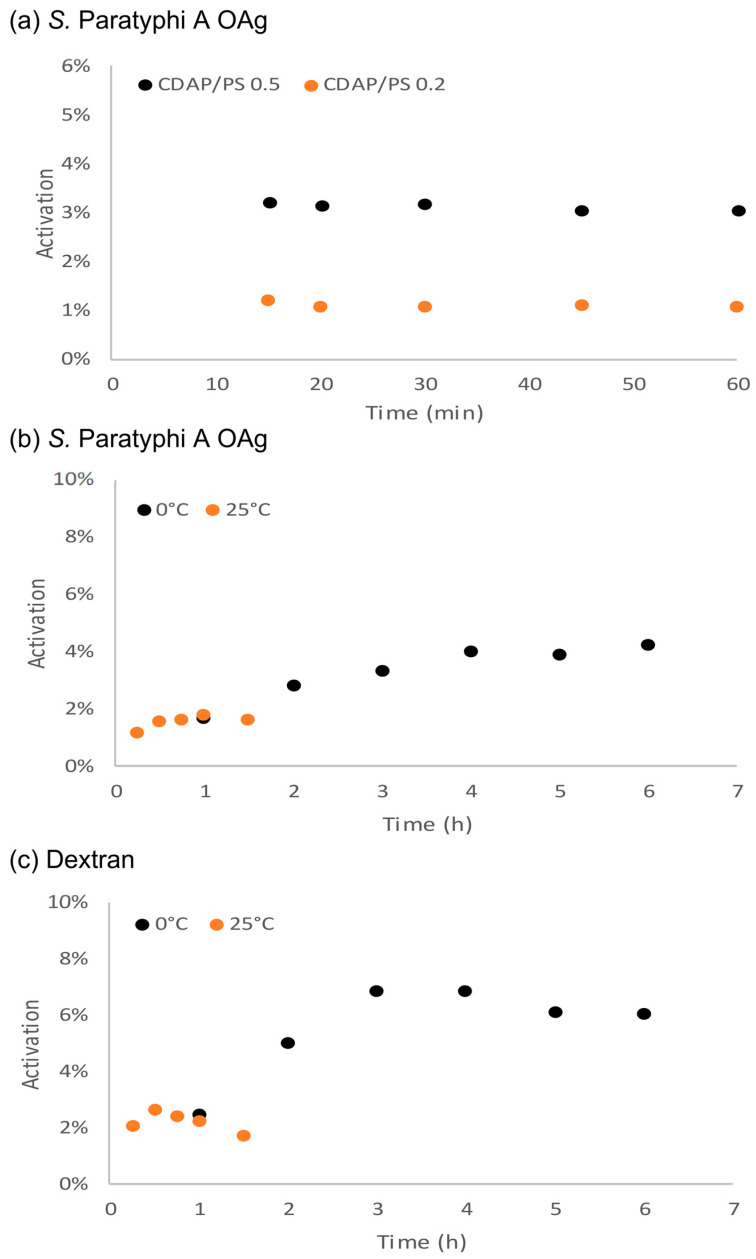
(**a**) CDAP activation kinetic of *S.* Paratyphi A OAg 1.1 mg/mL and CDAP/PS 0.5 or 0.2 *w*/*w* in DABCO buffer at pH 9 at 0 °C; (**b**) *S.* Paratyphi A OAg 4.4 mg/mL and CDAP/PS 0.5 *w*/*w* in Lutidine buffer at pH 7 at 0° or 25 °C; (**c**) activation kinetic of 4.4 mg/mL dextran CDAP/PS 0.5 *w*/*w* in Lutidine buffer at pH 7 at 0° or 25 °C. Activation values are reported in relation to monosaccharides.

Studies were then performed to verify if the different CDAP/PS ratios would result in different kinetic activations and therefore if the activation rate of the PS would depend on the CDAP concentration. *S.* Paratyphi OAg was activated with CDAP/PS *w*/*w* ratio 0.5 or 0.2 following the reaction up to 60 min (Figure 4a). The maximum activation was reached after 15 min in both conditions, and the resulting activated product was stable for at least 60 min. The results confirmed that a reduced CDAP/PS ratio resulted in a lower activation percentage, but this was not due to a slower kinetic of activation.

By working at pH 7, the activation kinetic at 0 °C was slower, and the maximum activation % was reached after 4 h (Figure 4b,c). By increasing the reaction temperature to 25 °C, the resulting PS activation % was lower than at 0 °C, very likely as a result of instability of the activated PS at higher temperatures. This was verified by working both with *S.* Paratyphi A OAg and 25 kDa dextran.

In the literature, CDAP activation protocols with or without salt are reported. We tested if the presence of 1 M NaCl in a DABCO buffer impacted the activation of *S.* Paratyphi A OAg. No significant differences were evidenced by performing the reaction with or without NaCl (three activation replicates were performed on different days) (Figure S3), even if a lower variability of activation degree was observed by performing the reaction in the presence of NaCl. In both conditions, the activation degree found was 8.8% and the coefficient of variation was 13% in water against 3% in the presence of 1 M NaCl.

Finally, the activation reaction with DABCO was verified at 25 °C, as the possibility to avoid pH adjustment is considered an even greater improvement when shorter reaction times are needed. *S.* Paratyphi A OAg and dextran were activated (PS 4.4 mg/mL in 1 M NaCl, CDAP/PS 0.5 *w*/*w*, in a DABCO buffer at pH 9 at 25 °C for 3 min), and the average activation values (three activation replicates on different days) were 4.8% (CV 38%) and 4.3% (CV 36%), respectively. Even if the absence of a pH adjustment makes the process easier, the activation degree obtained at 25 °C was about half with respect to the corresponding reaction performed at 0 °C for 15 min; moreover, the variability was higher for these conditions.

### 3.4. CDAP Activation of PS from Different Pathogens

Activation conditions selected for *S.* Paratyphi A OAg (Table 2) were extended to a panel of other neutral PSs (*Shigella* OAg, *Salmonella* OAg and *Klebsiella pneumoniae* OAg), obtaining activation degrees in the range of 1–6%, which are useful for conjugation (Table 2). Slightly different reaction conditions were applied to negatively charged PSs like *S. flexneri* 6 OAg and a panel of *K. pneumoniae* capsular polysaccharides (CPSs), resulting in similar activation degrees (Table 2).

When working with the zwitterionic *S. sonnei* OAg, the reaction was tested both at pH 9 and 7, as the concomitant presence of hydroxyl and amino groups in this PS could generate crosslinking among PS chains (at pH 7, amino groups are less reactive). Activation percentages were similar for both conditions tested (Table 2), with no crosslinking as verified by the HPLC-SEC analysis (Figure S4).

By using fully O-acetylated *S.* Typhi Vi PS, which do not have available hydroxyl groups, it was also verified that sugar carboxylic groups, which can be present on PSs, do not react with CDAP. The activation was tested both at 0 °C for 15 min and 25 °C for 3 min at pH 9 with PS 3 mg/mL and CDAP/PS 0.5 *w*/*w* ratio (1.5 mg/mL CDAP).

### 3.5. Parameters Affecting Conjugation of Activated PS to Protein

We then investigated the critical parameters that could impact the chemical linkage of the activated PS to the carrier protein. Conjugation temperature and time at two different pH values (7 and 9) were evaluated using *S.* Paratyphi A OAg and the CRM_197_ carrier protein as the model.

The temperature could affect the conjugation to the carrier protein both for the conjugation kinetic itself and for the stability of the activated PS. First, the stability of the CDAP-activated PS was evaluated at 5 °C and at 25 °C.

**Figure 5:**
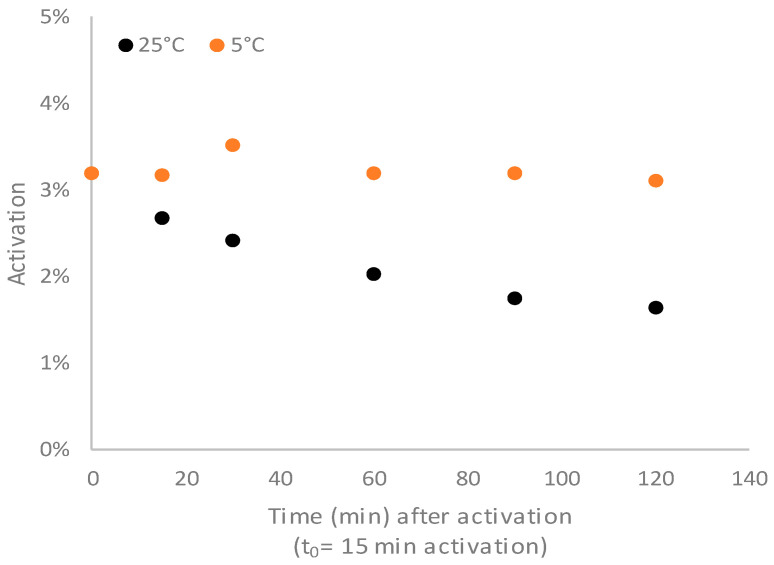
Activated PS stability evaluated for *S.* Paratyphi A OAg activated using the following conditions: 4.4 mg/mL PS and CDAP/PS 0.2 *w*/*w* (0.88 mg/mL CDAP) in a DABCO buffer at pH 9 at 0 °C. Activation values are reported related to monosaccharides.

The activated PS was stable at 5 °C for at least 2 h, while at 25 °C, degradation began after 15 min (Figure 5).

**Figure 6:**
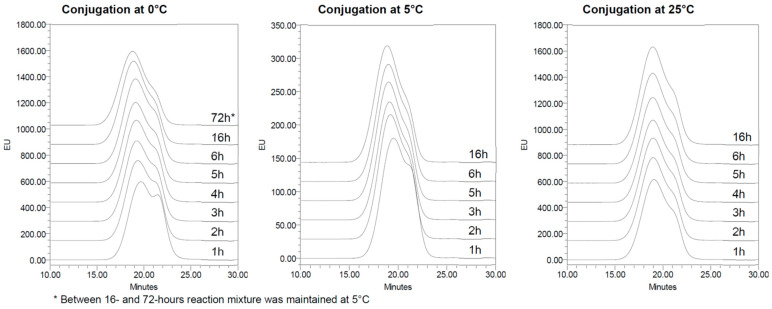
HPLC-SEC profiles of OAg-CRM_197_ conjugates (fluorescence emission ex280/em336) at different times during the conjugation step performed at 0–5–25 °C at pH 9. After activation at 0 °C for 15 min with a 0.5 CDAP/PS *w*/*w* ratio (2.2 mg/mL CDAP) in 50 mM DABCO at pH 9, 4.4 mg/mL OAg was conjugated with CRM_197_ at different temperatures using a PS/CRM_197_ 1/1 *w*/*w* ratio.

After having verified the stability of the CDAP-activated PS at different temperatures, the conjugation step of activated *S.* Paratyphi A OAg with CRM_197_ was performed at 0, 5 and 25 °C to understand the concomitant effect of activated PS stability and conjugation kinetics on the formation of the final conjugate. In all conditions, the resulting conjugation reaction was fast, with all CRM_197_ conjugated after 1 h. By increasing the reaction time, the conjugate MW increased as a result of increased PS loading on the protein or higher crosslinking. No major changes were observed after 16 h at 0 °C, 4 h at 5 °C or 2 h at 25 °C. After these times, all conjugates had a similar profile in HPLC-SEC (Figure 6, Table 3) independent from the temperature of the conjugation step.

At 25 °C, the conjugation reaction was faster, and after 2 h, it was completed, with no further significant cross-linking as evidenced from the MW reported in Table 3. At 0 °C, the conjugation reaction was slower, and MW differences became negligible after 16 h. From the comparison between different temperatures (2 h at 25 °C vs. 16 h at 0 °C), we conclude that 2 h at 25 °C or 16 h at 0 °C are needed to complete the conjugation reaction, resulting in conjugates with similar molecular size distributions.

**Figure 7:**
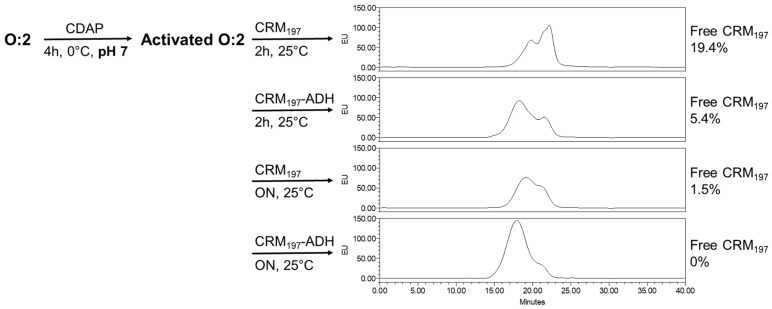
Activation performed with 4.4 mg/mL PS and CDAP/PS 0.5 (2.2 mg/mL CDAP) at pH 7 for 4 h at 0 °C; conjugation was then performed using PS/protein ratio of 1/1 using the conditions reported in the Figure.

We also verified that if the conjugation needs to be performed at pH 7 (e.g., for saccharide instability at pH 9), an overnight conjugation time is needed to avoid the presence of unreacted proteins. This is due to the lower reactivity of protein amino groups at pH 7 compared to pH 9. Indeed, by performing the conjugation with CRM_197_-ADH, bringing hydrazide groups that are more reactive at pH 7, free protein decreased from 19.4% to 5.4% after 2 h of reaction with CRM_197_ or CRM_197_-ADH, respectively (Figure 7).

By setting up a reaction mixture in a DABCO buffer at pH 9, without PS and with a CDAP concentration of 0.88 mg/mL, we verified that after 15 min at 0 °C (simulating the end of the activation step), unreacted CDAP, measured as the absorbance at 312 nm [13], was still present in the mixture (about 30–40% of its initial quantity) and could react with -OH groups on the protein during the following conjugation step. A conjugation reaction in the absence of PS was simulated, and after glycine quenching, a mass spectrum was recorded. In the mass spectrum, the main peak corresponding to the CRM_197_ protein was revealed together with a lower intensity peak corresponding to the protein derivatized with one glycine through CDAP chemistry (Figure S5). This confirmed that even in the worst case without PS, the protein may have only a limited modification on just one site for CRM_197_ in the condition tested.

### 3.6. Design of Experiment Characterization of Activation and Conjugation Reaction through CDAP Chemistry

Working with *S*. Paratyphi A OAg (O:2) and CRM_197_ as models, based on the preliminary studies performed, activation and conjugation times and temperatures were set as constant (the activation was performed at 0 °C for 15 min and the conjugation step at 25 °C for 2 h) to investigate the effect/synergy on activation/conjugation processes of three different critical factors identified: CDAP, PS concentrations and PS/CRM_197_ ratio. We studied the impact of such factors on the following responses:

- Activation rate of the PS (as % of activated sugar ring);
- Conjugated MW;
- Unconjugated CRM_197_;
- Unreacted PS;
- Conjugated saccharide to protein ratio.

To do this, we used a design of experiment (DoE) approach with the aim to fully understand the design space inside the specific ranges selected for each factor.

#### 3.6.1. Design of Experiment

A full two-level factorial design was used, with two replicates of factorial points and three replicates of center point. The plan ensured 81% statistical power to detect (5% alpha) signal/noise ratio of 1.5 in a full factorial model with two-way interactions. PS concentration, CDAP concentration and PS to CRM_197_ ratio in conjugation were evaluated in the range of 2.5–10 mg/mL, 1.25–5 mg/mL and 1–2 (*w*/*w*), respectively. Pearson’s correlation coefficients for different factor–response and response–response are reported in Figure S6.

**Figure 8:**
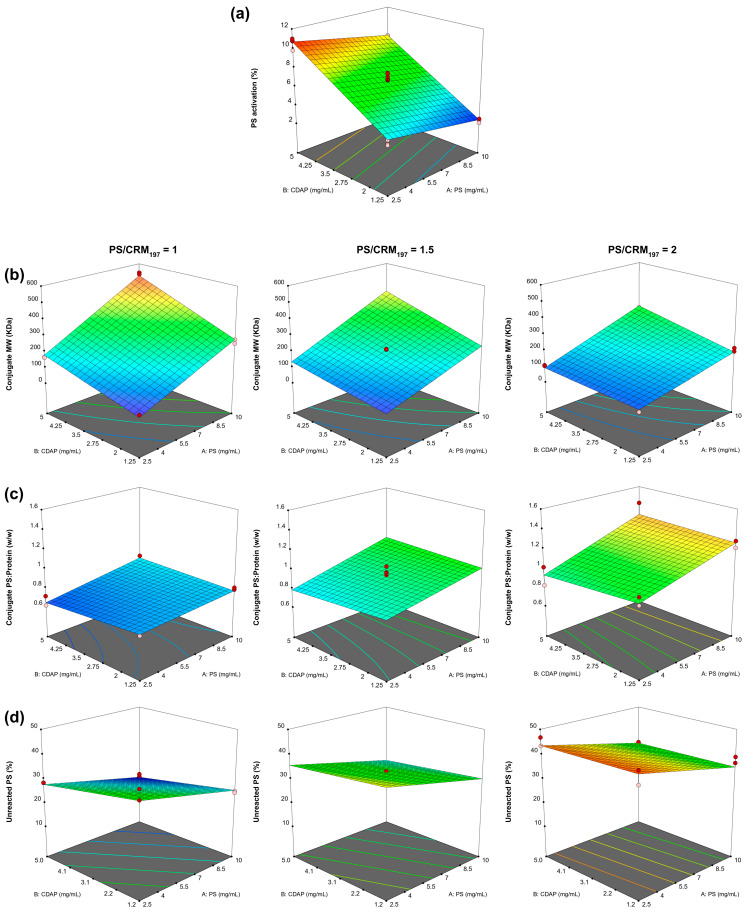
(**a**) Model for activation rate; (**b**) model for conjugate MW; (**c**) model for conjugate PS/protein ratio; and (**d**) model for unreacted PS.

**PS Activation model:** only PS and CDAP concentrations showed a significant impact without significant interaction terms (Figure S7). The model resulted in an adjusted R^2^ of 0.972 (Figure 8a), the residuals were normally distributed (Ryan–Joiner *p* > 0.100) and the lack of fit was not significant (*p* = 0.085). In particular, we demonstrated that by increasing the PS concentration, the activation degree slightly decreased. However, by increasing the CDAP concentration, higher activation values were obtained.

**Conjugate MW model:** PS, CDAP and CRM_197_ concentrations as well as the three two-factor interaction terms were significant (Figure S8). The model resulted in an adjusted R^2^ of 0.993 (Figure 8b), the residuals were normally distributed (Ryan–Joiner *p* > 0.100) and the lack of fit was significant (*p* = 0.003). Higher CDAP as well as higher PS concentrations generated conjugates with higher MWs, and the two factors had a positive interaction (the effect produced increasing one of them was enhanced when the other factor had a higher setting). The increase in the PS/CRM_197_ ratio in the reaction reduced the conjugate MW. Clearly, a higher PS concentration reduces the conjugate crosslinking given by the linkage of different proteins with the same PS chain.

**Conjugate PS/protein ratio model:** PS, CDAP and CRM_197_ concentrations as well as the two-factor interaction terms involving PS concentrations were significant (Figure S9). The model resulted in an adjusted R^2^ of 0.937 (Figure 8c), the residuals were normally distributed (Ryan–Joiner *p* > 0.100) and the lack of fit was not significant (*p =* 0.073). Higher PS concentrations as well as higher PS/CRM_197_ ratios in the conjugation mixture generated conjugates with higher PS/protein ratios. The two-factor interaction of these parameters had a positive interaction (the effect produced increasing one of them was enhanced when the other factor had a higher setting). Interestingly, the CDAP concentration had a minimal effect (even if statistically significant) on the conjugate PS/protein ratio.

**Unreacted PS model:** PS, CDAP and CRM_197_ concentrations as well as the two-factor interaction terms involving the PS concentration were significant (Figure S10). The model resulted in an adjusted R^2^ of 0.939 (Figure 8d), the residuals were normally distributed (Ryan–Joiner *p* > 0.100) and the lack of fit was significant (*p* = 0.027). Higher CDAP as well as higher PS concentrations decreased the unreacted PS, while the increase in the PS/CRM_197_ ratio in the reaction resulted in an increment of unreacted PS.

Also, the significant lack of fit suggests that higher order models would be necessary to analytically describe the conjugate MW and the unreacted PS, considering the R^2^ obtained, we valued these models as fit for our purpose to understand the responses’ dependency on the individual factors.

#### 3.6.2. Variability of CDAP Conjugation

Reproducibility and repeatability of the reaction were evaluated considering the replicates of the conjugation performed at the center of the design space and corresponding to the reaction conditions of PS and CDAP concentrations of 6.25 mg/mL and 3.125 mg/mL, respectively, and PS/protein ratio in conjugation of 1.5. ANOVA variance component analysis (general linear model with days as a random factor) was used to estimate the reproducibility (defined as the variability among different days), the repeatability (defined as the variability under the same operating conditions over a short interval of time) and the contribution of different days to the variability (Figure S11).

The results obtained, reported as the coefficient of variation (CV), are presented in Table 4; the CVs reported include both the reaction and analytical variability.

#### 3.6.3. Impact of Reaction Conditions and Conjugate Characteristics on the Immune Response Induced in Rabbits by Conjugates Generated through CDAP Chemistry

With the aim to explore the impact of reaction conditions and conjugate characteristics on immunogenicity, the O:2-CRM_197_ conjugates obtained with the DoE plan were tested in rabbits. Conjugates obtained using the same conditions as DoE replicates were pooled in order to have a total of nine groups for animal testing, corresponding to the eight factorial levels and the center point of the DoE scheme.

The impact of the DoE factors (e.g., PS concentration, CDAP concentration, PS to protein ratio in conjugation) on the humoral response was evaluated. The regression models directly correlating the conjugation reaction factors to the immunological characteristics of the conjugates (in terms of log EU/mL and log SBA titers) were built (Figure S12). Conjugates synthesized with lower O:2 to CRM_197_ ratios in the reaction led to higher IgG responses (at day 42) as well as higher SBA titers. Moreover, sera bactericidal activity increased for conjugates synthesized with higher PS concentrations in the reaction.

**Figure 9:**
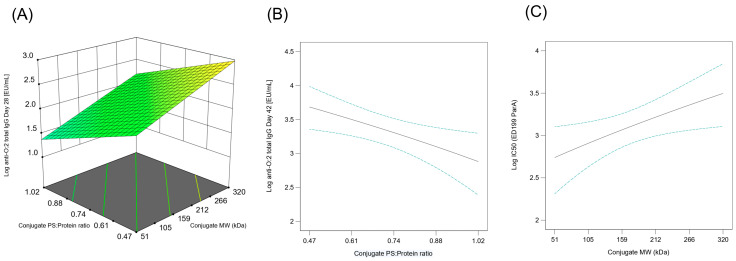
Impact on the immune response in rabbits induced by O:2-CRM_197_ conjugates differing for PS/protein ratio and MW. (**A**) Model for the impact of the conjugate O:2/CRM_197_ *w*/*w* and MW on log EU/mL at day 28; (**B**) model for the impact of the conjugate O:2/CRM_197_ *w*/*w* on log EU/mL at day 42; (**C**) model for the impact of the conjugate MW on log SBA IC50 at day 42.

The correlations of conjugate properties (MW and O:2/CRM_197_ *w*/*w* ratio) with immunological responses are of great interest. Multiple linear regression analyses were performed (Figure S13), and significant models were found for the anti-O:2 IgG at both day 28 (with no significant lack of fit, *p* = 0.347) and 42 (with no significant lack of fit, *p* = 0.325) and for the bactericidal titers at day 42 (with no significant lack of fit, *p* = 0.393). In particular, we demonstrated that the anti-O:2 IgG response in rabbits decreased by increasing the conjugate O:2/CRM_197_ *w*/*w* ratio (*p* = 0.016 and *p* = 0.010, respectively) in the range of 0.47–1.02 both at days 28 and 42, while with the increase in the conjugate MW, an increase in the IgG response at day 28 (*p* = 0.037) and in the bactericidal activity at day 42 (*p* = 0.022) was observed (Figure 9).

## 4. Discussion

Chemical conjugation is a well-established technology for carbohydrate-based vaccines. Here, with the aim to identify an easy conjugation approach generically applicable to PSs with different structural features and supporting the rapid development of high-valency conjugates, CDAP chemistry was selected and further optimized. Starting from the published procedure [19,21], the reaction for PS activation was improved by using DABCO as a buffer. DABCO has a high buffer capacity at pH 9 and allows for avoiding pH adjustment during PS activation. Moreover, the use of a low temperature (0 °C) allowed us to increase the reaction time from a few minutes to longer times, making the activation step easier to control and more consistent (the cyanylated PS results were stable for hours in the reaction conditions). In parallel, a novel analytical method based on HPLC-SEC was put in place for fast and easy determination of the PS activation degree. A single analysis not only allows us to quantify PS activation degree but also to check for PS integrity and lack of aggregation or degradation potentially happening during the reaction.

PS activation was mainly impacted by the absolute concentration of CDAP and not by CDAP to PS ratio (Figure 8a and Figure S7), and the CDAP concentration in a reaction can be easily adjusted to obtain the percent of activation desired. By working with dextrans and *S.* Paratyphi A OAg as model PSs, optimal reaction conditions were identified to provide optimal activation degrees to guarantee efficient conjugation with no major impacts on PS epitopes, which is relevant for preserving good immunogenicity. Importantly, the reaction worked successfully on a large panel of different PSs (made of neutral, negatively charged and zwitterionic sugars), with limited adjustments (Table 2). The CDAP chemistry can be easily used for synthesis of glycoconjugates as well as to produce tools for chemical and immuno assays.

CDAP activation is performed at pH 9, but the use of a basic pH might not be compatible with some PSs, affecting their structural stability. Moreover, many PSs are decorated with O-acetyl groups [40], which can play a major role in the immune response of the corresponding glycoconjugates (e.g., *Salmonella typhi* Vi [41] and meningococcal serogroup A [42,43,44]). Because O-acetyl groups are base-labile, a basic environment is potentially able to affect the O-acetylation level of the saccharide, causing O-acetyl release in the solution. For these reasons, we also identified optimal reaction conditions to perform the activation step with CDAP at pH 7, identifying Lutidine HCl as an appropriate buffer system.

**Figure 10:**
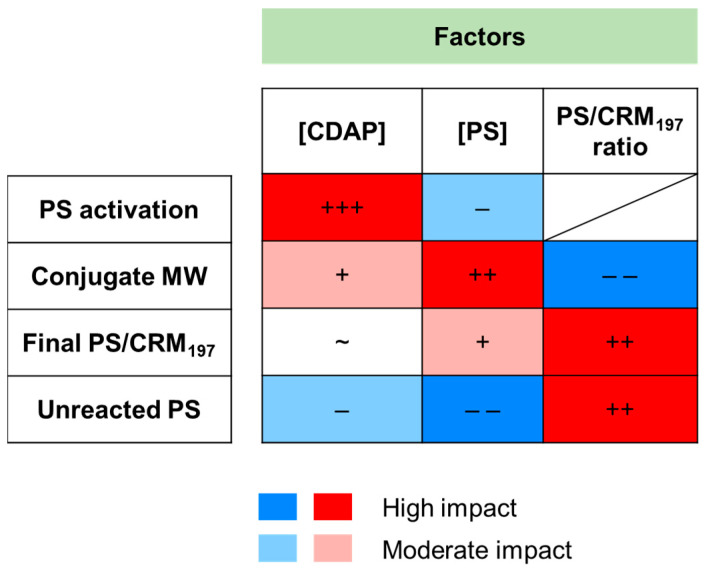
Graph summarizing the impact of DoE factors (CDAP concentration, PS concentration and PS/CRM_197_ ratio) evaluated during *S.* Paratyphi A OAg conjugation to CRM_197_ by CDAP chemistry on PS activation degree, conjugate MW, PS to CRM_197_ ratio of the conjugate and unreacted PS.

By using *S*. Paratyphi A OAg as a model, we identified the correlation among conjugate attributes and the different critical factors evaluated during CDAP conjugation as well as the possible correlation among the different conjugate attributes (Figure 10 and Figure S6). The models found for *S.* Paratyphi A OAg and CRM_197_ were not numerically predictive for other PSs or proteins but will simplify identification of conditions to be used with other systems, as the effects of reaction factors on conjugate parameters should be similar.

Once a specific PS of a given chain length and a certain carrier protein are selected, other parameters such as PS to protein ratio, percent of activated groups along the PS chain and conjugate molecular size need to be investigated for optimal design of the corresponding conjugate. Here, we showed how a simple plan of conjugation tests can be performed to identify critical parameters impacting conjugate characteristics, defining the space in which the different conjugation parameters can be changed without impacting the features of the wanted conjugate, reducing the probability of failure and driving the reaction towards a conjugate with the desired and optimal characteristics for a good immune response. The values of reproducibility found (2–6%) confirmed that the process is very robust and consistent. Also, conditions to have efficient conjugation, minimizing the percent of unreacted PS, can be easily identified.

By testing the different *S*. Paratyphi A O:2-CRM_197_ conjugates generated through the DoE in rabbits, we were able to correlate the different conjugation reaction conditions (i.e., CDAP and PS concentration and starting O:2/CRM_197_ *w*/*w* ratio) and the final conjugate characteristics (i.e., conjugate MW and PS/protein *w*/*w* ratio) with the immunogenicity. Rabbits were chosen considering that a full human dose and volume can be tested in this animal model. By applying linear regression models for interpretation of the immunological results, the statistical power of the study was increased. In fact, while the Kruskal–Wallis test was able to detect just one significant difference among the nine groups tested (only for ELISA results at day 28), the linear regression analysis demonstrated that different conjugation reaction conditions and final conjugate characteristics have an impact on immunogenicity. In particular, in rabbits, in the range of conjugate O:2/CRM_197_ *w*/*w* ratio of 1.02 ÷ 0.47, a lower O:2/CRM_197_ ratio increased the anti-O:2 IgG response both after the first and second injection, while a higher conjugate MW (in the range of 51 kDa ÷ 302 kDa) induced a higher anti-IgG response at day 28 and SBA titers at day 42.

In conclusion, we have identified the conditions to make the random conjugation approach based on CDAP chemistry more consistent. CDAP chemistry can be generally applied to different PSs and proteins as it utilizes common functional groups and is very fast, as no isolation of activated intermediates or use of linkers is required. The work done here has allowed us to understand how to consistently control the PS activation degree, which is important to not alter PS epitopes, resulting in final conjugates with desired characteristics for a good immune response. *S.* Paratyphi A OAg was used as a model, but the results obtained can be easily extended to the conjugation of other PSs and support rapid and efficient development of high-valency conjugates.

## Figures and Tables

**Table 1:** Activation degree for dextran and *S.* Paratyphi A OAg obtained using different experimental conditions.

	DABCO Buffer, pH 9 *				
CDAP/sugar (*w*/*w*)	0.2	0.32	0.5	1	2
PS mg/mL in activation	4.4	4.4	4.4	4.3	3.8
CDAP mg/mL in activation	0.88	1.4	2.2	4.3	7.7
Temperature and time	0 °C × 15 min				
Dextran activation % sugar rings/total	NA	6.3	8.1 **	11.4	14.6
OAg activation % sugar rings/total	3.6	4.4	5.8 **	9.8	NA

* DABCO concentrations were 50 mM for CDAP/PS among 0.2 to 1 *w*/*w* ratio and 100 mM for CDAP/PS 2 *w*/*w* ratio (for buffering capacity). ** Average of multiple tests. NA: data not available.

**Table 2:** CDAP activation applied to different PSs.

PS	Structure *	Nature	Activation Conditions	Activation		PS MW (KDa)
				% Sugar Ring	% RU	
*S.* Paratyphi A OAg	α-Par*p-*(1→3) ¬ α-Glc*p-*(1→6)¬ [→2)-α-Man*p*-(1→4)-α-Rha*p*-(1→3)-α-Gal-(1→]_n_ [29]	N	4.4 mg/mL PS, CDAP/PS 0.2 *w*/*w* (0.88 mg/mL CDAP), DABCO at pH 9, 15 min	3.7	18.3	100–16
*S.* Typhimurium OAg	α-Abe*p-*(1→3)¬ α-Glc*p-*(1→4 or 6) ¬ [→2)-α-Man*p*-(1→4)-α-Rha*p*-(1→3)-α-Gal-(1→]_n_ [30]	N		1.9	9.3	33.5
*S.* Enteritidis OAg	α-Tyv*p-*(1→3) ¬ α-Glc*p-*(1→4 or 6) ¬ [→2)-α-Man*p*-(1→4)-α-Rha*p*-(1→3)-α-Gal-(1→]_n_ [31]	N		2	10.2	92.1–26.7
*Klebsiella p.* O1 OAg	[→3)-α-Gal*p*-(1→3)-β-Gal*p*-(1→]_m_-[→3)-β-Gal*f*-(1→3)-α-Gal*p*-(1→]_n_ [32]	N		5	10.0	22.8
*Klebsiella p.* O2a OAg	[→3)-β-Gal*f*-(1→3)-α-Gal*p*-(1→]_n_ [32]	N		5.5	11.1	10.7
*S. flexneri* 1b OAg	α-Glc*p-*(1→4) ¬ [→2)-α-Rha*p*-(1→2)-α-Rha*p*-(1→3)-α-Rha*p*-(1→3)-β-Glc*p*NAc-(1→]_n_ [33]	N		1.9	9.7	13.5
*S. flexneri* 2a OAg	α-Glc*p-*(1→4) ¬ [→2)-α-Rha*p*-(1→2)-α-Rha*p*-(1→3)-α-Rha*p*-(1→3)-β-Glc*p*NAc-(1→]_n_ [33]	N		2.9	14.4	41.5–13.4
*S. flexneri* 3a OAg	α-Glc*p-*(1→3) ¬ [→2)-α-Rha*p*-(1→2)-α-Rha*p*-(1→3)-α-Rha*p*-(1→3)-β-Glc*p*NAc-(1→]_n_ [33]	N		2	10.1	69–15.7
*S. flexneri* 4a OAg	α-Glc*p-*(1→6) ¬ [→2)-α-Rha*p*-(1→2)-α-Rha*p*-(1→3)-α-Rha*p*-(1→3)-β-Glc*p*NAc-(1→]_n_ [33]	N		2.6	12.9	17.2
*S. flexneri* 5b OAg	α-Glc*p-*(1→3)¬ α-Glc*p-*(1→3)¬ [→2)-α-Rha*p*-(1→2)-α-Rha*p*-(1→3)-α-Rha*p*-(1→3)-β-Glc*p*NAc-(1→]_n_ [33]	N		1.4	8.3	74.7–17.8
*S. flexneri* X OAg	α-Glc*p-*(1→3)¬ [→2)-α-Rha*p*-(1→2)-α-Rha*p*-(1→3)-α-Rha*p*-(1→3)-β-Glc*p*NAc-(1→]_n_ [33]	N		0.9	4.3	66.9–16.4
*S. flexneri* Y OAg	[→2)-α-Rha*p*-(1→2)-α-Rha*p*-(1→3)-α-Rha*p*-(1→3)-β-Glc*p*NAc-(1→]_n_ [33]	N		1.1	4.5	60–15
*Klebsiella p.* *CPS K2*	α-Glc*p*A*-*(1→3)¬ [→3)-β-Glc*p*-(1→4)-β-Man*p*-(1→4)-α-Glc*p*-(1→]_n_ [34]	NC	1.8 mg/mL PS, CDAP/PS 1 *w*/*w* (1.8 mg/mL CDAP), DABCO at pH 9, 15 min	5.2	20.6	256 **
*Klebsiella p.* *CPS K25*	β-Glc*p-*(1→2)-β-Glc*p*A*-*(1→4)¬ [→4)-β-Glc*p*-(1→3)-β-Gal*p*-(1→]_n_ [35]	NC		6.2	24.7	312 **
*Klebsiella p.CPS K62*	α-Man*p-*(1→3)¬ [→4)-α-Glc*p*-(1→2)-β-Glc*p*A-(1→2)-α-Man*p*-(1→3)-β-Gal*p*-(1→]_n_ [36]	NC		4.3	21.5	335 **
*Klebsiella p.* *CPS K64*	(6,4-O-Pyr)-β-Glc*p*-(1→2)¬ [→4)-α-Glc*p*A-(1→3)-α-Man*p*-(1→3)-β-Glc*p*-(1→4)-α-Man*p*-(1→]_n_ [37] α-Rha*p*-(1→3)⌋	NC		6.1	36.5	105 **
*S. flexneri* 6 OAg	[→2)-α-Rha*p*-(1→2)-α-Rha*p*-(1→4)-β-Gal*p*A-(1→3)-β-Gal*p*NAc-(1→]_n_ [38]	NC		1.9	7.8	22.9
*S. sonnei* OAg	[→4)-α-Alt*p*NAcA-(1→3)-β-Fuc*p*NAc4N-(1→]_n_ [39]	ZW	1.1 mg/mL PS, CDAP/PS 0.5 *w*/*w* (0.55 mg/mL CDAP), DABCO at pH 9, 15 min	2.5	5.0	38.6
*S. sonnei* OAg	[→4)-α-Alt*p*NAcA-(1→3)-β-Fuc*p*NAc4N-(1→]_n_ [39]	ZW	1.1 mg/mL PS, CDAP/PS 0.5 *w*/*w* (0.55 mg/mL CDAP), Lutidine at pH 7, 4 h	3.7	7.4	38.6

N: neutral, NC: negatively charged; ZW: zwitterionic. * In the reported structures, eventual O-acetylation positions are omitted. ** CPS size before activation was reduced by sonication. Reported MW is in reference to treated PS.

**Table 3:** Conjugated MW with respect to the conjugation reaction time at different temperatures.

Time (h)	MW (kDa)		
	0 °C	5 °C	25 °C
1	140	138	212
2	176	176	226
3	191	187	228
4	196	195	229
5	208	198	232
6	208	203	234
16	242	221	229
72 *	259	-	-

* Between 16- and 72-h reaction mixture was maintained at 5 °C.

**Table 4:** Reproducibility and repeatability.

	Activation %	Conjugate PS/Protein	Conjugate MW	Unreacted PS
CV_Day_	3.6%	5.2%	-	-
CV_Error_	4.7%	3.6%	2.2%	3.2%
CV_Total_	5.9%	6.3%	2.2%	3.2%

## Data Availability

The authors declare that data are contained within the article and in the Supplementary Materials.

## Supplementary Materials

The following supporting information can be downloaded at: https://www.mdpi.com/article/10.3390/vaccines12070707/s1, Figure S1: confidence intervals for average PS activation degree between reactions in presence of DMAP or DABCO; Figure S2: MS spectra of reaction products of cyclodextrin; Figure S3: confidence intervals for average O:2 OAg activation degree between reactions with or without 1M NaCl; Figure S4: comparison among HPLC-SEC profiles of *S. sonnei* OAg, and *S. sonnei* OAg-ADH with activation performed at pH 7 or 9; Figure S5: CRM_197_ MS spectra prior and post CDAP treatment; Figure S6: Pearson’s correlation coefficients for factors and responses in DoE runs; Figure S7: PS activation model; Figure S8: Conjugate MW model; Figure S9: Conjugate PS/protein model; Figure S10: Unreacted Polysaccharide model; Figure S11: Variance component analysis for PS activation, PS/Protein ratio, conjugate MW and unreacted PS; Figure S12: Impact of the conjugation factors on the humoral response; Figure S13: Multiple linear regression analysis for Log EU/mL at day 28, for Log EU/mL at day 42 and for Log SBA IC50 at day 42; Figure S14: DOE runs schematic representation. Table S1: Peak intensity ratios from MS spectra of reaction products of cyclodextrin; Table S2: DOE runs conditions.
