# Supplementary material for: Modeling 1-Cyano-4-Dimethylaminopyridine Tetrafluoroborate (CDAP) Chemistry to Design Glycoconjugate Vaccines with Desired Structural and Immunological Characteristics
Source: Vaccines (Basel). 2024 Jun 24;12(7):707. doi: 10.3390/vaccines12070707 (PMC11281720; doi:10.3390/vaccines12070707)
Supplement: Supplementary file 1 [file vaccines-12-00707-s001.zip › vaccines-2993786-supplementary.pdf]

Supplementary Materials

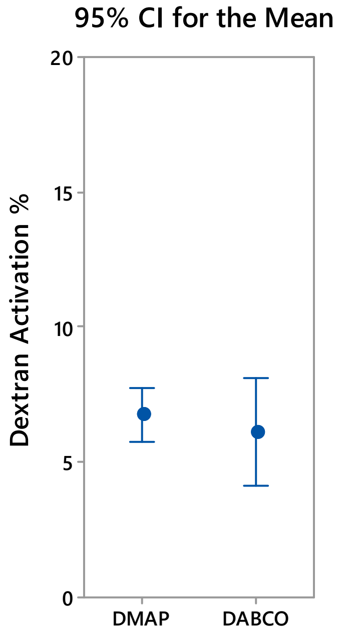

Figure S1: comparison of confidence intervals for average PS (25 KDa dextran) activation degree between reactions conducted in presence of DMAP or DABCO (4.4 mg/mL saccharide concentration, CDAP/PS 0.5 w/w ratio (2.2 mg/mL CDAP), pH 9, 0 °C for 15 minutes).

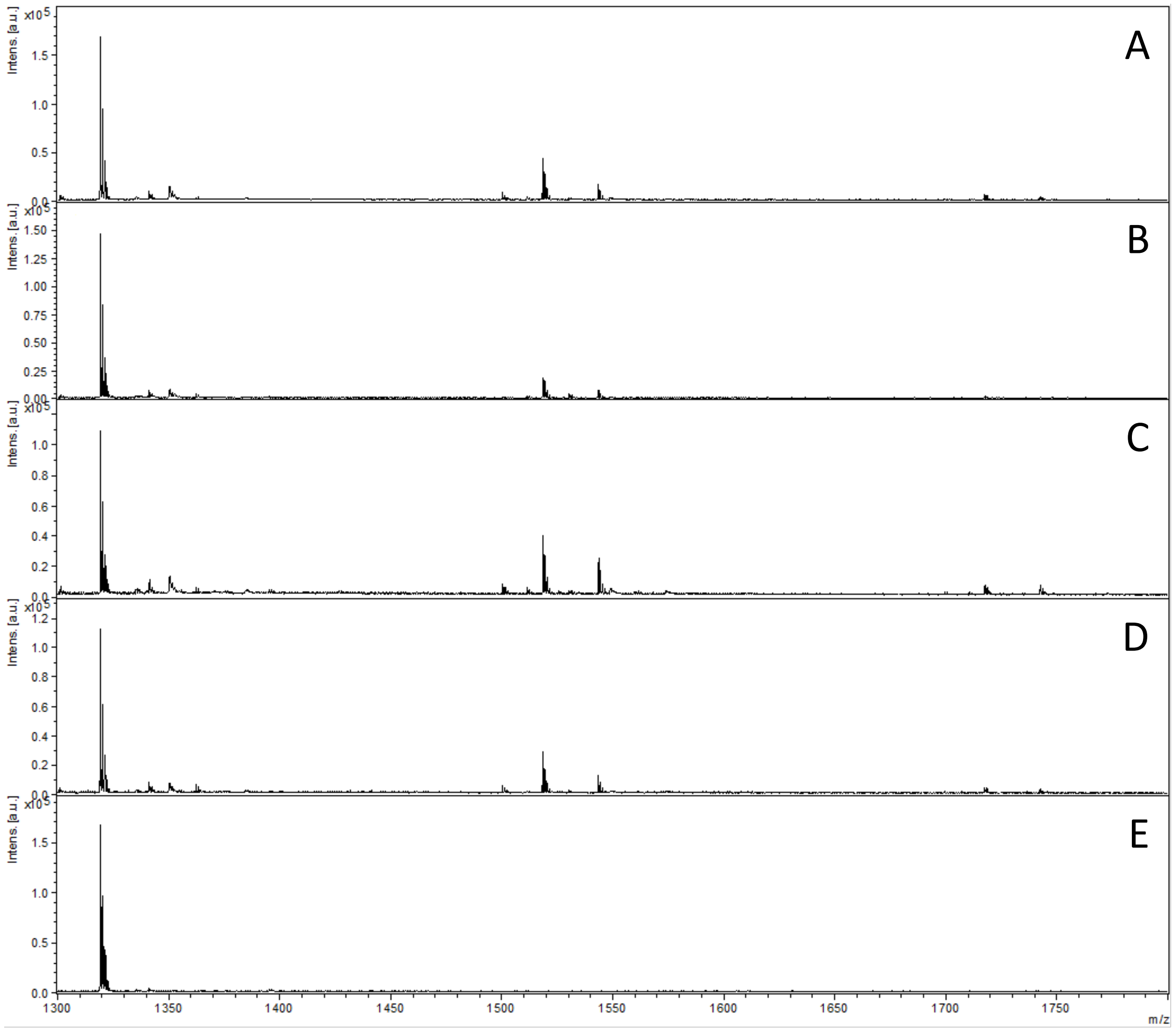

Figure S2: MS spectra of reaction products of cyclodextrin 4.4 mg/mL: **a)** Lutidine buffer pH 7 with CDAP/CD 0.5 w/w (2.2 mg/mL CDAP); **b)** DABCO buffer pH 9 with CDAP/CD 0.2 w/w (0.88 mg/mL CDAP); **c)** DABCO buffer pH 9 with CDAP/CD 0.5 w/w (2.2 mg/mL CDAP); **d)** DMAP/NaOH pH 9 with CDAP/CD 0.5 w/w (2.2 mg/mL CDAP); **e)** underivatized cyclodextrin.

Table S1: Peak intensity ratios from MS spectra of reaction products of cyclodextrin.

**Peak Intensity ratio**	**DABCO CDAP/CD 0.5 w/w**	**DABCO CDAP/CD 0.2 w/w**	**DMAP/NaOH CDAP/CD 0.5 w/w**	**Lutidine CDAP/CD 0.5 w/w**
$[CD\ ADH\ Na]^+ / [CD\ Na]^+$	0.36	0.17	0.24	0.25
$[CD\ ADH_2\ Na]^+ / [CD\ Na]^+$	0.07	-	0.04	0.04
$[CD\ IMC\ Na]^+ / [CD\ Na]^+$	0.06	0.03	0.06	0.03
$[CD\ ADH\ IMC\ Na]^+ / [CD\ ADH\ Na]^+$	0.60	0.30	0.46	0.40
$[CD\ ADH_2\ IMC\ Na]^+ / [CD\ ADH_2\ Na]^+$	0.94	-	0.79	0.69

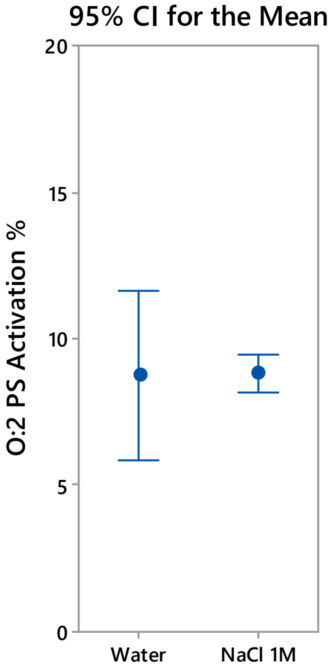

Figure S3: comparison of confidence intervals (n=3) for average O:2 OAg activation degree between reactions conducted with or without 1M NaCl, with 4.4 mg/mL PS starting concentration, CDAP/PS w/w 0.5 (2.2 mg/mL CDAP) at pH 9 in DABCO buffer.

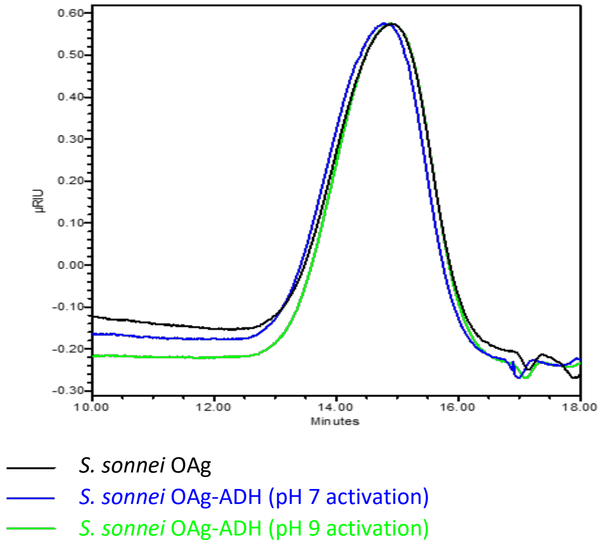

Figure S4: comparison among HPLC-SEC profiles (dRI) of *S. sonnei* OAg, and *S. sonnei* OAg-ADH with activation performed at pH 7 or 9.

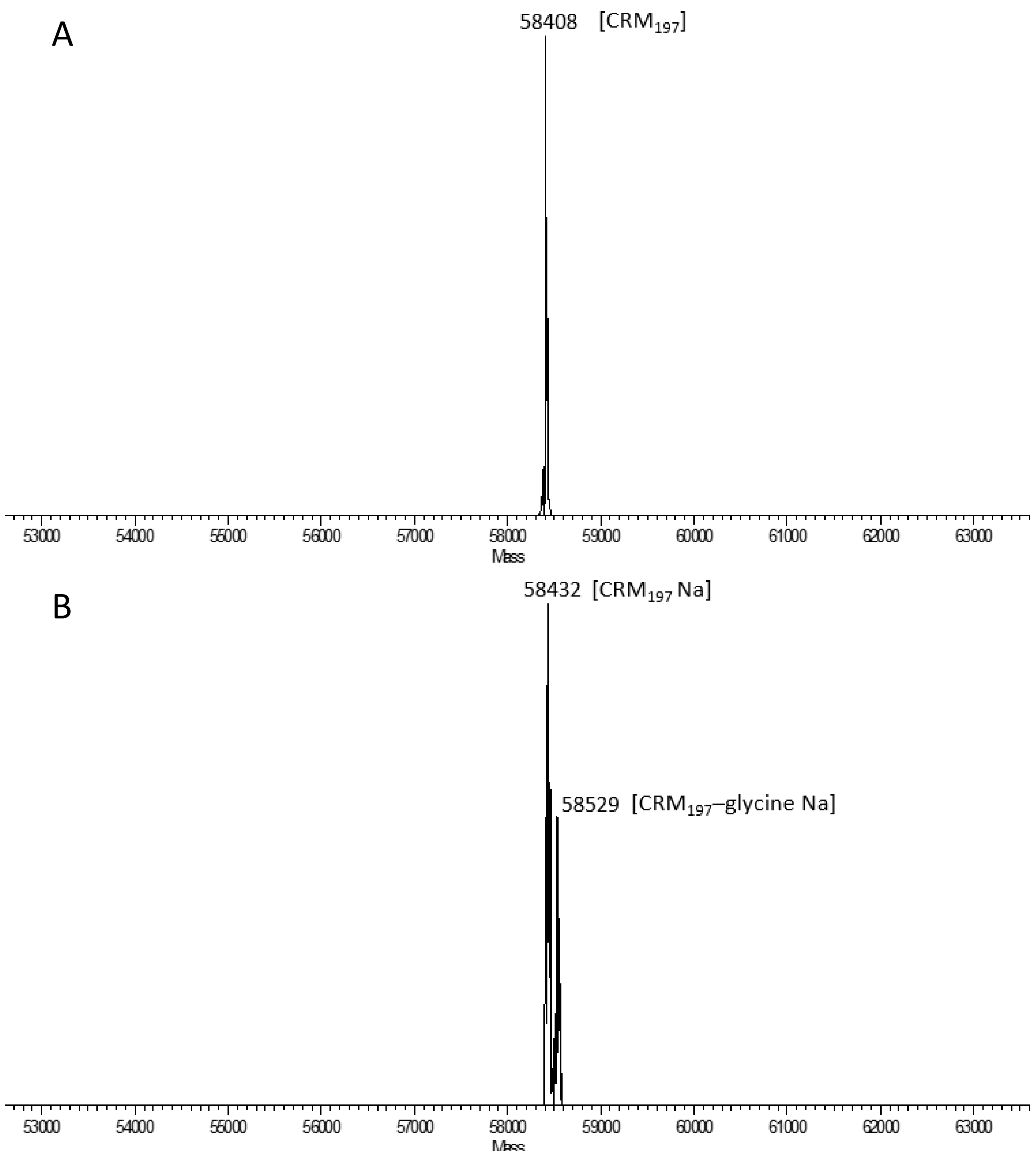

Figure S5: CRM_{197} MS spectra prior (a) and post (b) CDAP treatment. A reaction was performed without PS (pH 9, DABCO 50 mM, CDAP 3.6 mg/mL, 0°C, 15 min); CRM_{197} was then added (3.6 mg/mL) and after 2h at RT the reaction was quenched with glycine 1M solution. The final solution was desalted with PD10 column in ammonium acetate and analyzed by MS, in comparison to CRM_{197} solution not treated with CDAP.

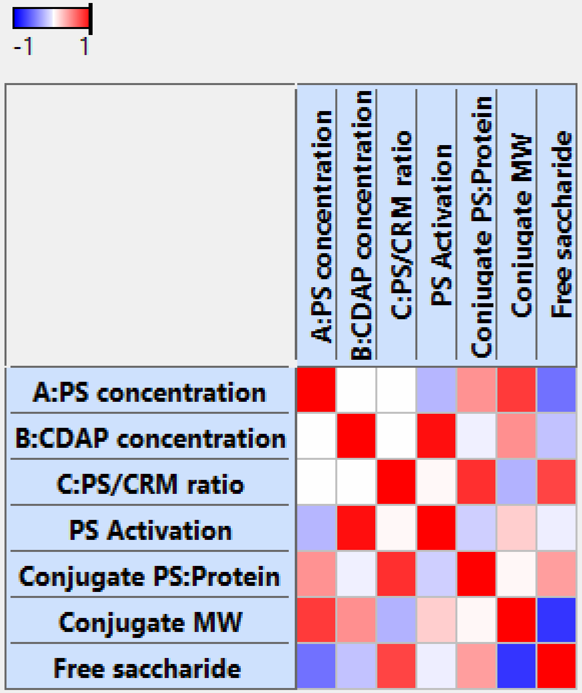

	A:PS concentration	B:CDAP concentration	C:PS/CRM ratio	PS Activation	Conjugate PS:Protein	Conjugate MW	Free saccharide
A:PS concentration	1.000	0.000	0.000	-0.282	0.423	0.769	-0.553
B:CDAP concentration	0.000	1.000	0.000	0.944	-0.054	0.434	-0.234
C:PS/CRM ratio	0.000	0.000	1.000	0.021	0.811	-0.296	0.729
PS Activation	-0.282	0.944	0.021	1.000	-0.183	0.185	-0.061
Conjugate PS:Protein	0.423	-0.054	0.811	-0.183	1.000	0.027	0.375
Conjugate MW	0.769	0.434	-0.296	0.185	0.027	1.000	-0.794
Free saccharide	-0.553	-0.234	0.729	-0.061	0.375	-0.794	1.000

Figure S6: Pearson's correlation coefficients for factors and responses in DoE runs.

ANOVA for Linear model

Response 2: PS Activation

Source	Sum of Squares	df	Mean Square	F-value	p-value	
Block	0.6880	3	0.2293			
**Model**	177.38	2	88.69	414.49	< 0.0001	significant
A-PS	14.53	1	14.53	67.89	< 0.0001	
B-CDAP	162.85	1	162.85	761.09	< 0.0001	
**Residual**	4.71	22	0.2140			
Lack of Fit	3.87	14	0.2764	2.64	0.0852	not significant
Pure Error	0.8372	8	0.1047			
**Cor Total**	182.78	27				

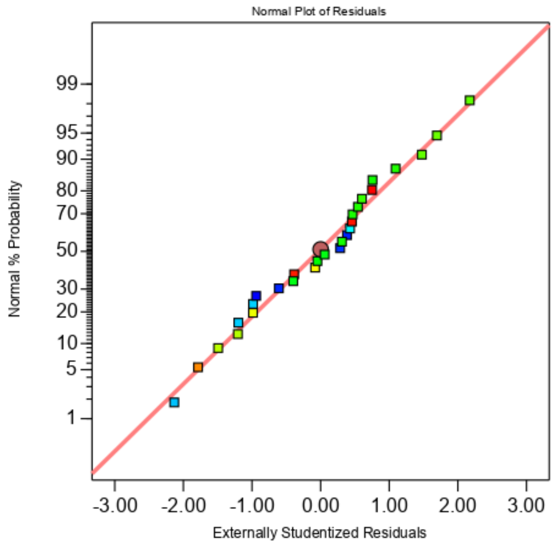

Fit Statistics

**Std. Dev.**	0.4626		**R^2**	0.9741
**Mean**	6.55		**Adjusted R^2**	0.9718
**C.V. %**	7.07		**Predicted R^2**	0.9577
			**Adeq Precision**	40.7551

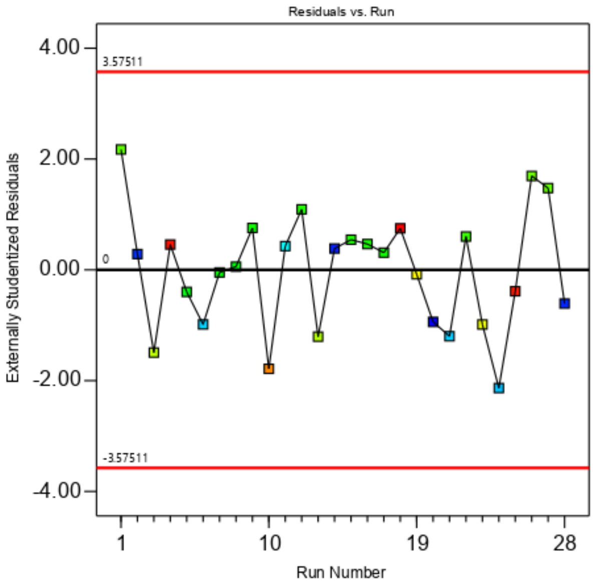

Figure S7: PS activation model.

ANOVA

	Source	Sum of Squares	df	Mean Square	F-value	p-value	
	Block	1946.11	3	648.70			
	**Model**	3.483E+05	6	58057.25	531.96	< 0.0001	significant
	A-PS	2.084E+05	1	2.084E+05	1909.42	< 0.0001	
	B-CDAP	66306.25	1	66306.25	607.54	< 0.0001	
	C-PS/CRM	30800.25	1	30800.25	282.21	< 0.0001	
	AB	11342.25	1	11342.25	103.92	< 0.0001	
	AC	15500.25	1	15500.25	142.02	< 0.0001	
	BC	16002.25	1	16002.25	146.62	< 0.0001	
	**Residual**	1964.50	18	109.14			
	Lack of Fit	1794.50	10	179.45	8.44	0.0030	significant
	Pure Error	170.00	8	21.25			
	**Cor Total**	3.523E+05	27				

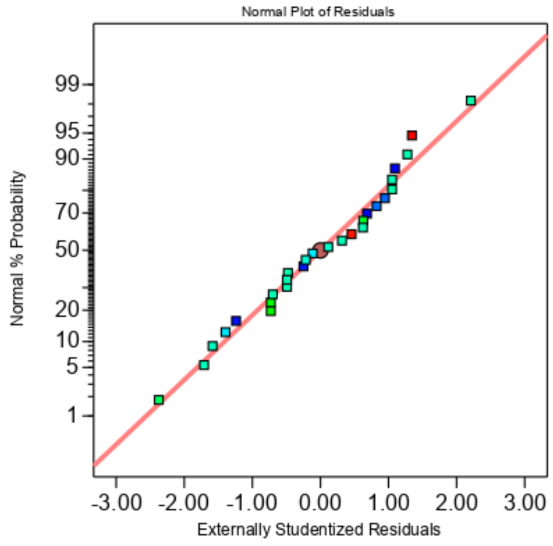

Fit Statistics

	**Std. Dev.**	10.45		**R^2**	0.9944
	**Mean**	209.32		**Adjusted R^2**	0.9925
	**C.V. %**	4.99		**Predicted R^2**	0.9841
				**Adeq Precision**	78.0614

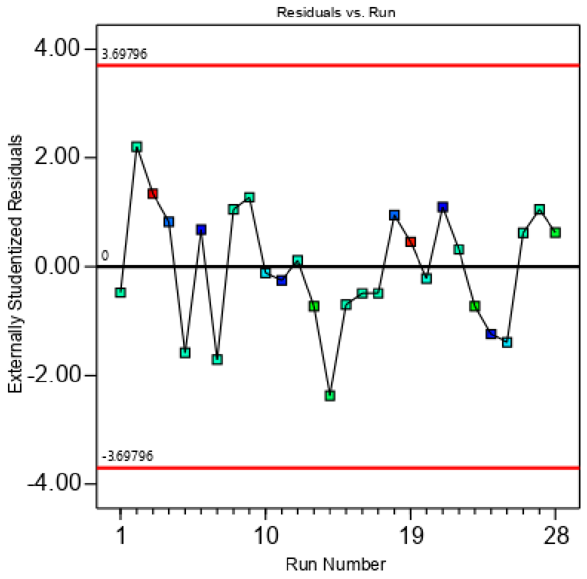

Figure S8: Conjugate MW model.

ANOVA

Source	Sum of Squares	df	Mean Square	F-value	p-value	
Block	0.0489	3	0.0163			
**Model**	0.8368	5	0.1674	72.09	< 0.0001	significant
A-PS	0.1661	1	0.1661	71.52	< 0.0001	
B-CDAP	0.0028	1	0.0028	1.19	0.2895	
C-PS/CRM	0.6123	1	0.6123	263.74	< 0.0001	
AB	0.0127	1	0.0127	5.45	0.0307	
AC	0.0431	1	0.0431	18.55	0.0004	
**Residual**	0.0441	19	0.0023			
Lack of Fit	0.0352	11	0.0032	2.86	0.0730	not significant
Pure Error	0.0089	8	0.0011			
**Cor Total**	0.9298	27				

Fit Statistics

**Std. Dev.**	0.0482	**R^2**	0.9499
**Mean**	0.9268	**Adjusted R^2**	0.9367
**C.V. %**	5.20	**Predicted R^2**	0.8556
		**Adeq Precision**	26.9777

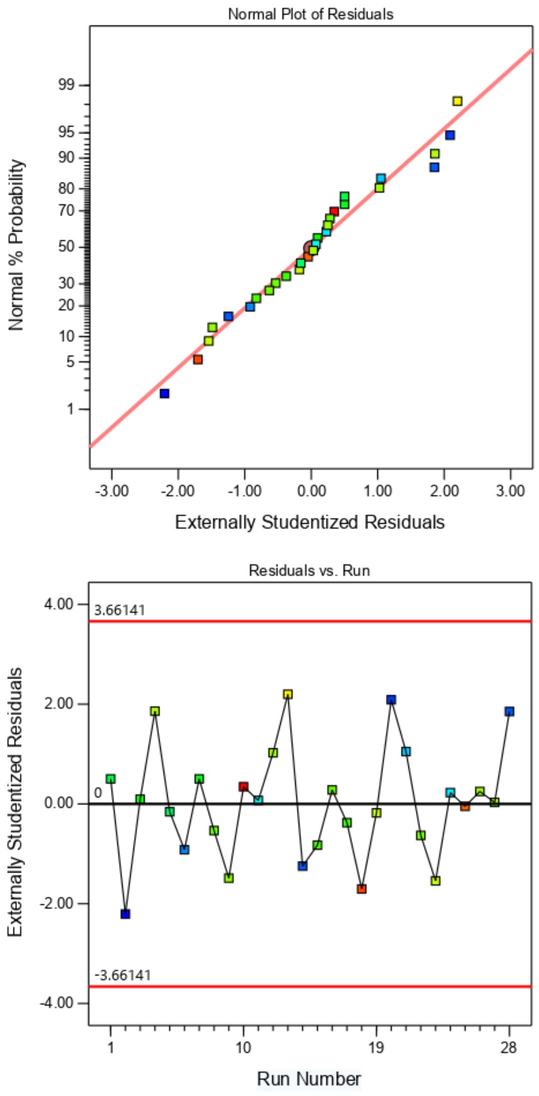

Figure S9: Conjugate PS/protein model.

ANOVA

Source	Sum of Squares	df	Mean Square	F-value	p-value	
Block	33.02	3	11.01			
**Model**	1122.32	4	280.58	93.43	< 0.0001	significant
A-PS	371.56	1	371.56	123.72	< 0.0001	
B-CDAP	66.79	1	66.79	22.24	0.0001	
C-PS/CRM	645.18	1	645.18	214.84	< 0.0001	
BC	38.80	1	38.80	12.92	0.0018	
**Residual**	60.06	20	3.00			
Lack of Fit	51.69	12	4.31	4.11	0.0266	significant
Pure Error	8.38	8	1.05			
**Cor Total**	1215.40	27				

Fit Statistics

**Std. Dev.**	1.73	**R^2**	0.9492
**Mean**	32.91	**Adjusted R^2**	0.9390
**C.V. %**	5.27	**Predicted R^2**	0.8921
		**Adeq Precision**	29.4705

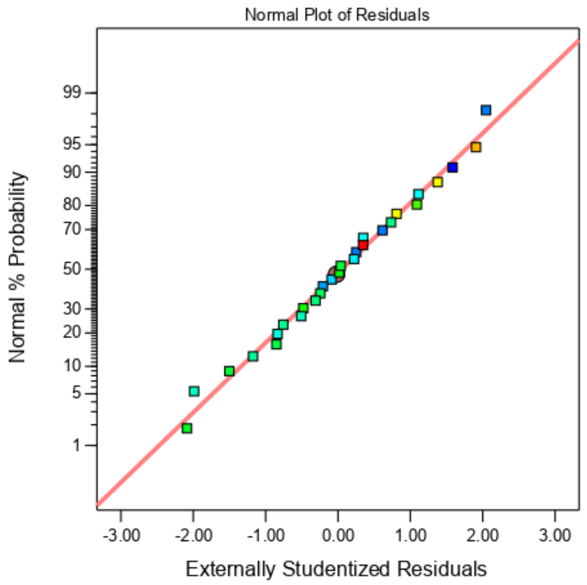

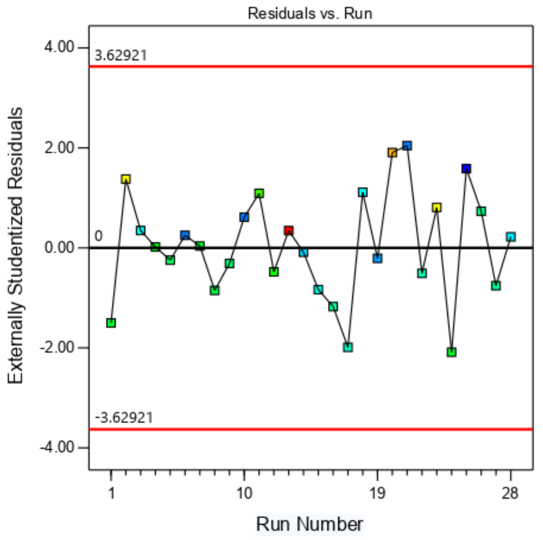

Figure S10: Unreacted Polysaccharide model.

General Linear Model: Activation % versus Day

Method

Factor coding (-1; 0; +1)

Factor Information

Factor	Type	Levels	Values
Day	Random	4	Day 1; Day 2; Day 3; Day 4

Analysis of Variance

Source	DF	Adj SS	Adj MS	F-Value	P-Value
Day	3	0.8470	0.2823	2.70	0.116
Error	8	0.8372	0.1047		
Total	11	1.6842			

Variance Components, using Adjusted SS

Source	Variance	% of Total	StDev	% of Total
Day	0.0592224	36.14%	0.243357	60.12%
Error	0.104652	63.86%	0.323500	79.91%
Total	0.163875		0.404814	

General Linear Model: PS:protein versus Day

Method

Factor coding (-1; 0; +1)

Factor Information

Factor	Type	Levels	Values
Day	Random	4	Day 1; Day 2; Day 3; Day 4

Analysis of Variance

Source	DF	Adj SS	Adj MS	F-Value	P-Value
Day	3	0.024033	0.008011	7.17	0.012
Error	8	0.008933	0.001117		
Total	11	0.032967			

Variance Components, using Adjusted SS

Source	Variance	% of Total	StDev	% of Total
Day	0.0022981	67.30%	0.0479390	82.04%
Error	0.0011167	32.70%	0.0334166	57.18%
Total	0.0034148		0.0584364	

General Linear Model: Conjugate MW versus Day

Method

Factor coding (-1; 0; +1)

Factor Information

Factor	Type	Levels	Values
Day	Random	4	Day 1; Day 2; Day 3; Day 4

Analysis of Variance

Source	DF	Adj SS	Adj MS	F-Value	P-Value
Day	3	22.92	7.639	0.36	0.784
Error	8	170.00	21.250		
Total	11	192.92			

Variance Components, using Adjusted SS

Source	Variance	% of Total	StDev	% of Total
Day	-4.53704*	0.00%	0.00000	0.00%
Error	21.25	100.00%	4.60977	100.00%
Total	21.25		4.60977	

** Value is negative, and is estimated by zero.*

General Linear Model: Unreacted PS versus Day

Method

Factor coding (-1; 0; +1)

Factor Information

Factor	Type	Levels	Values
Day	Random	4	Day 1; Day 2; Day 3; Day 4

Analysis of Variance

Source	DF	Adj SS	Adj MS	F-Value	P-Value
Day	3	2.383	0.7942	0.76	0.548
Error	8	8.377	1.0471		
Total	11	10.759			

Variance Components, using Adjusted SS

Source	Variance	% of Total	StDev	% of Total
Day	-0.0842965*	0.00%	0.00000	0.00%
Error	1.04707	100.00%	1.02326	100.00%
Total	1.04707		1.02326	

** Value is negative, and is estimated by zero.*

Figure S11: Variance component analysis for PS activation, PS/Protein ratio, conjugate MW and unreacted PS. Average values: Activation = 6.85%; Conjugate PS/protein = 0.922; Conjugate MW = 208.5 KDa; Unreacted PS = 31.9%.

A) ANOVA and surface plot for Log EU/mL at day 28 model

Source	Sum of Squares	df	Mean Square	F-value	p-value	
**Model**	14.54	3	4.85	5.55	0.0018	significant
B-CDAP	1.90	1	1.90	2.18	0.1449	
C-O2 to CRM	8.99	1	8.99	10.28	0.0020	
BC	3.65	1	3.65	4.18	0.0448	
**Residual**	59.44	68	0.8741			
Lack of Fit	0.5049	1	0.5049	0.5740	0.4513	not significant
Pure Error	58.93	67	0.8796			
**Cor Total**	73.98	71				

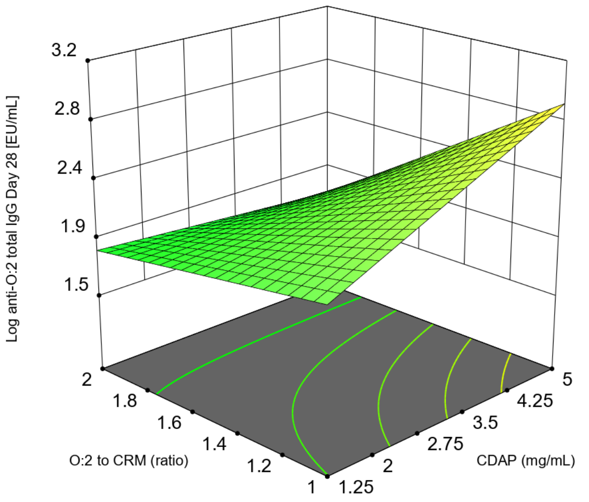

B) ANOVA and plot for Log EU/mL at day 42 model

Source	Sum of Squares	df	Mean Square	F-value	p-value	
**Model**	119.97	1	119.97	6.08	0.0161	significant
C-O2 to CRM	119.97	1	119.97	6.08	0.0161	
**Residual**	1381.62	70	19.74			
Lack of Fit	31.87	1	31.87	1.63	0.2061	not significant
Pure Error	1349.74	69	19.56			
**Cor Total**	1501.58	71				

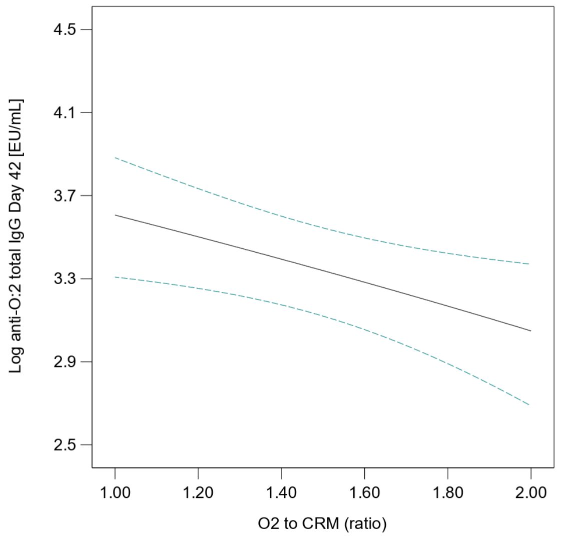

C) ANOVA and surface plot for Log IC50 at day 42 model

Source	Sum of Squares	df	Mean Square	F-value	p-value	
**Model**	108.14	2	54.07	4.15	0.0198	significant
A-O2	59.90	1	59.90	4.60	0.0355	
C-O2 to CRM	48.24	1	48.24	3.70	0.0584	
**Residual**	898.61	69	13.02			
Lack of Fit	5.64	2	2.82	0.2116	0.8098	not significant
Pure Error	892.97	67	13.33			
**Cor Total**	1006.75	71				

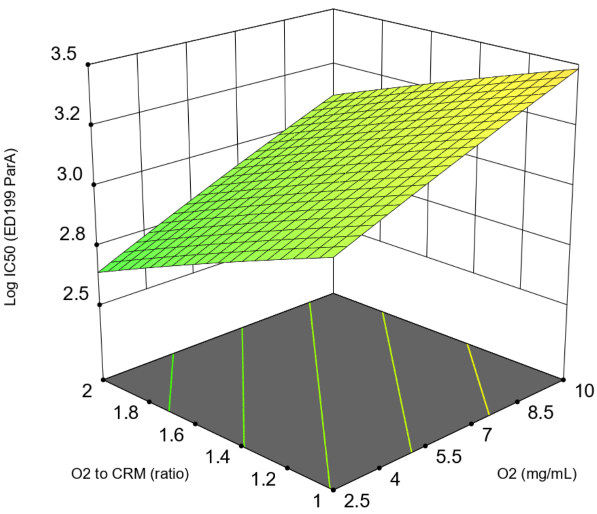

Figure S12: Impact of the conjugation factors (PS concentration, CDAP concentration, PS to protein ratio in conjugation) on the humoral response.

A) ANOVA and residual normality plot for Log EU/mL at day 28 model

Source	Sum of Squares	df	Mean Square	F-value	p-value	
**Model**	10.94	2	5.47	5.99	0.0040	significant
A-Conjugate MW	4.14	1	4.14	4.54	0.0368	
B-Conjugate PS:Protein ratio	5.60	1	5.60	6.13	0.0158	
**Residual**	63.04	69	0.9137			
Lack of Fit	6.20	6	1.03	1.15	0.3470	not significant
Pure Error	56.84	63	0.9023			
**Cor Total**	73.98	71				

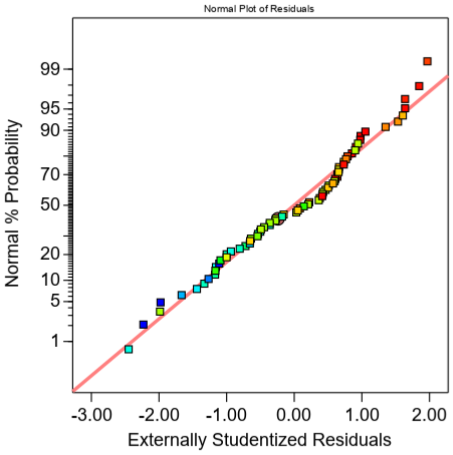

B) ANOVA and residual normality plot for Log EU/mL at day 42 model

Source	Sum of Squares	df	Mean Square	F-value	p-value	
**Model**	135.80	1	135.80	6.96	0.0103	significant
B-Conjugate PS:Protein ratio	135.80	1	135.80	6.96	0.0103	
**Residual**	1365.78	70	19.51			
Lack of Fit	158.76	7	22.68	1.18	0.3249	not significant
Pure Error	1207.02	63	19.16			
**Cor Total**	1501.58	71				

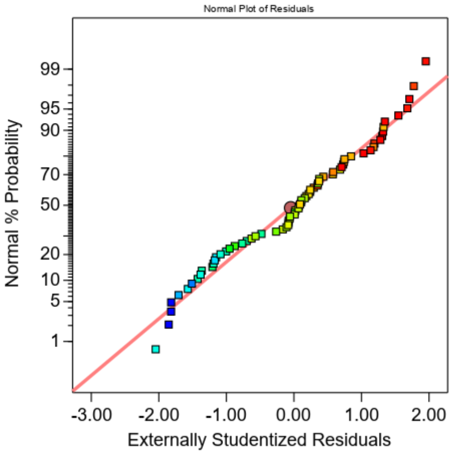

C) ANOVA and residual normality plot for Log IC50 at day 42

Source	Sum of Squares	df	Mean Square	F-value	p-value	
**Model**	97.26	1	97.26	5.46	0.0223	significant
A-Conjugate MW	97.26	1	97.26	5.46	0.0223	
**Residual**	1246.21	70	17.80			
Lack of Fit	132.39	7	18.91	1.07	0.3933	not significant
Pure Error	1113.82	63	17.68			
**Cor Total**	1343.48	71				

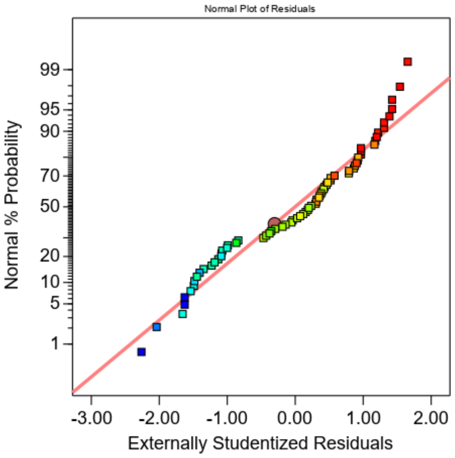

Figure S13: Multiple linear regression analysis for A) Impact of the conjugate O:2/CRM$_{197}$ w/w and MW on Log EU/mL at day 28; B) Impact of the conjugate O:2/CRM$_{197}$ w/w on Log EU/mL at day 42; C) Impact of the conjugate MW on Log SBA IC50 at day 42.

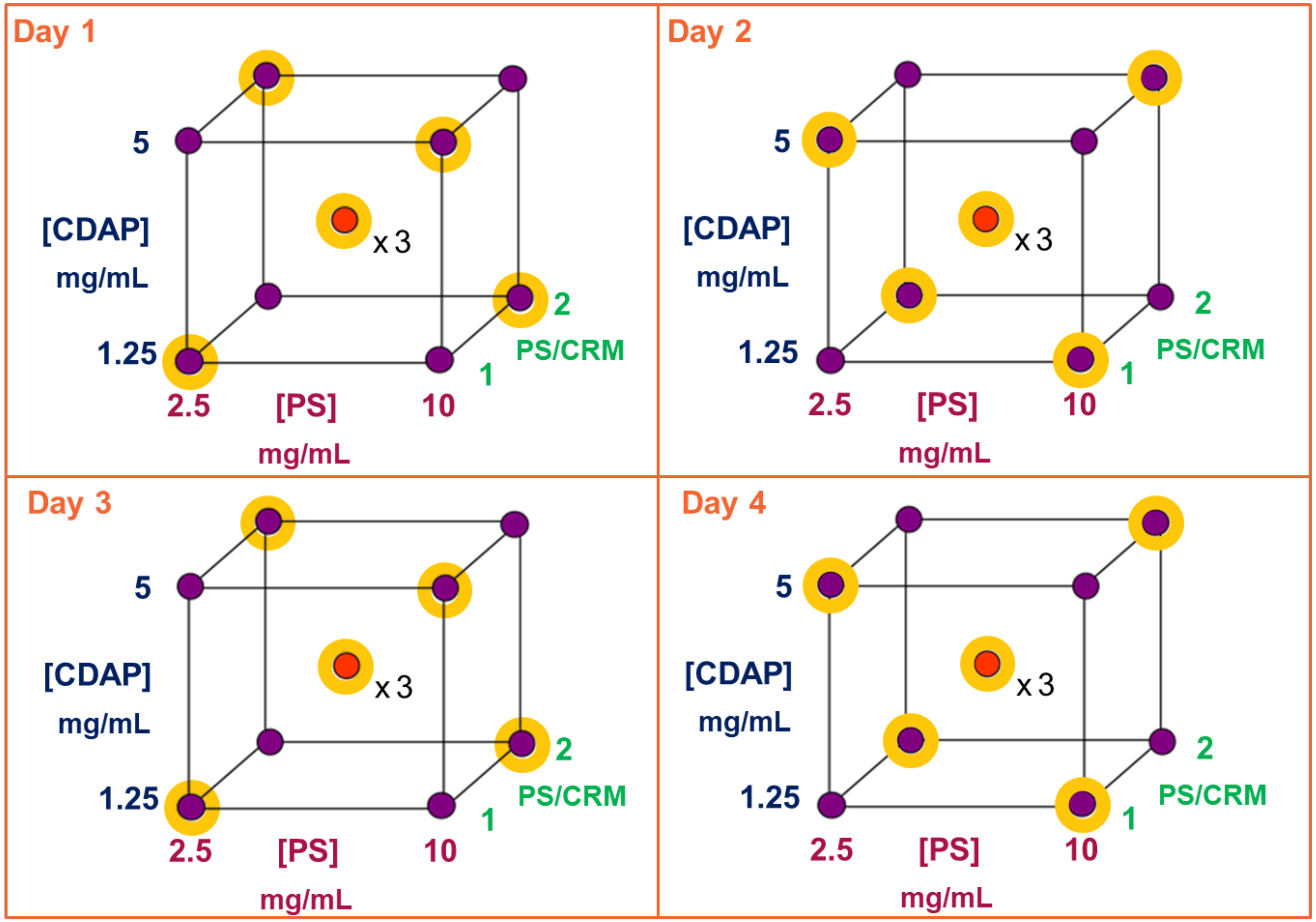

Figure S14: DOE runs schematic representation.

Table S2: DOE runs conditions.

Block	Run	Space Type	Factor 1 A:PS mg/mL	Factor 2 B:CDAP mg/mL	Factor 3 C:CRM$_{197}$ PS/CRM$_{197}$	Calculation sugar ring µmol/mL	Calculation CDAP µmol/mL	Calculation CDAP/ sugar ring w/w	Calculation CDAP/ sugar ring mol ratio	Quantities in run *CDAP100* solution µL	Quantities in run *DABCO5* solution µL	Quantities in run *CRM20* solution µL
Day 1	1	Center	6.25	3.125	1.5	41.3	13.3	0.50	0.322	31.25	233	167
	2	Factorial	10	1.25	2	66.1	5.3	0.13	0.080	12.5	200	200
	3	Factorial	10	5	1	66.1	21.3	0.50	0.322	50	0	400
	4	Factorial	2.5	5	2	16.5	21.3	2.00	1.288	50	350	50
	5	Center	6.25	3.125	1.5	41.3	13.3	0.50	0.322	31.25	233	167
	6	Factorial	2.5	1.25	1	16.5	5.3	0.50	0.321	12.5	300	100
	7	Center	6.25	3.125	1.5	41.3	13.3	0.50	0.322	31.25	233	167
Day 2	8	Center	6.25	3.125	1.5	41.3	13.3	0.50	0.322	31.25	233	167
	9	Center	6.25	3.125	1.5	41.3	13.3	0.50	0.322	31.25	233	167
	10	Factorial	2.5	5	1	16.5	21.3	2.00	1.288	50	300	100
	11	Factorial	2.5	1.25	2	16.5	5.3	0.50	0.321	12.5	350	50
	12	Center	6.25	3.125	1.5	41.3	13.3	0.50	0.322	31.25	233	167
	13	Factorial	10	5	2	66.1	21.3	0.50	0.322	50	200	200
	14	Factorial	10	1.25	1	66.1	5.3	0.13	0.080	12.5	0	400
Day 3	15	Center	6.25	3.125	1.5	41.3	13.3	0.50	0.322	31.25	233	167
	16	Center	6.25	3.125	1.5	41.3	13.3	0.50	0.322	31.25	233	167
	17	Center	6.25	3.125	1.5	41.3	13.3	0.50	0.322	31.25	233	167
	18	Factorial	2.5	5	2	16.5	21.3	2.00	1.288	50	350	50
	19	Factorial	10	5	1	66.1	21.3	0.50	0.322	50	0	400
	20	Factorial	10	1.25	2	66.1	5.3	0.13	0.080	12.5	200	200
	21	Factorial	2.5	1.25	1	16.5	5.3	0.50	0.321	12.5	300	100
Day 4	22	Center	6.25	3.125	1.5	41.3	13.3	0.50	0.322	31.25	233	167
	23	Factorial	10	5	2	66.1	21.3	0.50	0.322	50	200	200
	24	Factorial	2.5	1.25	2	16.5	5.3	0.50	0.321	12.5	350	50
	25	Factorial	2.5	5	1	16.5	21.3	2.00	1.288	50	300	100
	26	Center	6.25	3.125	1.5	41.3	13.3	0.50	0.322	31.25	233	167
	27	Center	6.25	3.125	1.5	41.3	13.3	0.50	0.322	31.25	233	167
	28	Factorial	10	1.25	1	66.1	5.3	0.13	0.080	12.5	0	400
